# Capsular polysaccharide of *Mycoplasma mycoides* subsp. *capri* contributes to phenotypic diversity promoting distinctive immune responses

**DOI:** 10.1371/journal.ppat.1013386

**Published:** 2025-12-12

**Authors:** Thatcha Yimthin, Marilou Bourgeon, Jing Zhang, Lukas Eggerschwiler, Raphael Siegenthaler, Elise Schieck, Sergi Torres-Puig, Nicolas Ruggli, Thomas Démoulins, Joerg Jores

**Affiliations:** 1 Department of Infectious Diseases and Pathology, Institute of Veterinary Bacteriology, Vetsuisse Faculty, University of Bern, Bern, Switzerland; 2 Graduate School for Cellular and Biomedical Sciences (GCB), University of Bern, Bern, Switzerland; 3 Research Contracts Animals Group, Agroscope, Posieux, Switzerland; 4 International Livestock Research Institute (ILRI), Nairobi, Kenya; 5 Department of Infectious Diseases and Pathobiology, Institute of Virology and Immunology IVI, Vetsuisse Faculty, University of Bern, Mittelhäusern, Switzerland; 6 Multidisciplinary Center for Infectious Diseases (MCID), University of Bern, Bern, Switzerland; Miami University, UNITED STATES OF AMERICA

## Abstract

*Mollicutes* are minute cell wall less bacteria encompassing important pathogens. We show that pathogenic *Mycoplasma mycoides* subsp. *capri* (*Mmc*) can switch expression of capsular polysaccharide (CPS), which creates phenotypic diversity and has dramatic repercussions on immune responses. To characterize the immune responses, we employed the highly virulent *Mmc* strain GM12 as well as an engineered CPS-deficient mutant in a set of assays employing primary blood cells from its native caprine host and cattle. Primary blood cells stimulated with GM12 showed only very moderate effects on cell viability as well as activation marker expression supporting an immunological furtiveness-like lifestyle. Interestingly, GM12 showed the capacity to survive and resist inside monocyte-derived macrophages (MDMs), which fosters dissemination and persistence in the host. Stimulation with the CPS-deficient mutant which exposes surface proteins including lipoproteins, increased cell death, strongly suppressed expression of major histocompatibility complex on antigen-presenting cells and induced secretion of several pro-inflammatory cytokines/chemokines which is a clinical hallmark in infected animals. Moreover, the CPS-deficient mutant elicited inflammatory cell death in MDMs. In conclusion, we showed that *Mmc* can switch the expression of CPS, which leads to different immunological trajectories paving the way for clinical disease, dissemination and persistence in the host.

## Author summary

Mycoplasmas are cell-wall deficient minute bacteria that encompass important human and animal pathogens. Despite the absence of classical virulence factors such as toxins in many pathogenic mycoplasmas, the later can cause severe infectious diseases, including life-threatening pleuropneumonia or septicemia. The clinical disease is often associated with massive inflammation of affected tissues. It is well known that mycoplasmas can vary their surface protein expression, which contributes to phenotypic diversity and immune evasion. Several pathogenic mycoplasmas are known to possess a capsule. This article reports the ability of *Mycoplasma mycoides* subsp. *capri*, to switch the expression of capsular polysaccharide on and off, which profoundly influences immune responses in its natural ruminant hosts. The presence of capsulated as well as capsule-deficient mycoplasmas in the host adds another layer to phenotypic diversity and promotes different host immune responses that likely foster the dissemination, inflammation and pathogen persistence commonly observed during infections with mycoplasmas.

## Introduction

Bacteria belonging to the class *Mollicutes* are the smallest and simplest bacteria that can be cultivated in axenic media. They are characterized by the absence of a cell wall and a minute genome, because of reductive evolution. The *Mollicutes* species *Mycoplasma mycoides* is a deadly ruminant pathogen of utmost importance. Its subspecies *M. mycoides* subsp. *mycoides* (*Mmm*) causes contagious bovine pleuropneumonia in cattle [1], whereas its other subspecies *M. mycoides* subsp. *capri* (*Mmc*) causes different diseases in small ruminants such as caprine Mastitis, Arthritis, Keratitis, Pneumonia, and Septicemia syndrome (MAKePS) [2]. *Mmc* GM12 (GM12), a strain isolated from a large disease outbreak in the USA in 1980 [3,4], is highly virulent and deadly in goats. Experimental infections using this strain caused infected animals to reach endpoint criteria within six days [5]. Several candidate virulence factors such as capsular polysaccharide (CPS) [6,7], different lipoproteins [8,9], the *Mycoplasma* Ig binding (MIB)-*Mycoplasma* Ig protease (MIP) system [10], or the hydrogen peroxide release [11] have been reported. Capsular polysaccharide of GM12 is composed by β-(1→6)-galactofuranose. It has been reported for *Mmm*, that cells of the same strain can be encapsulated or be devoid of a capsule, while the latter *Mmm* cells shed the same polysaccharide that otherwise constitutes a capsule [12,13]. Moreover, it was reported for *Mmm* that the free exopolysaccharide isolated from a bacterial culture possess anti-inflammatory properties [14]. By using synthetic genomics tools, the UDP-galactopyranose mutase encoding gene *glf* was deleted in GM12::YCpMmyc1.1 [15]. The mutant GM12::YCpMmyc1.1-Δ*glf* was shown to be deficient of CPS using galactofuranose-specific antibodies and stains. Subsequent experiments employing this mutant have shown that β-(1→6)-galactofuranose contributes to membrane stability and that the CPS conceals adhesins *in vitro* [16]. Later, it was shown in an *in vivo* experiment employing the native host, that CPS in *Mmc* is a true virulence factor [17].

We have recently reported a peripheral blood mononuclear cells (PBMCs) based *ex vivo* platform to decipher bovine immune responses [18,19], in order to get insight into the host-pathogen interactions of bovine pathogens such as *Mycoplasmopsis bovis*. The purpose of the present study is to decipher host-*Mmc* interaction with a focus on immune cells to better understand the interplay between the pathogen and the host. A better knowledge of pathogenicity mechanisms is likely to foster the development of a rationale vaccine for *Mycoplasma mycoides* [20]. Therefore, we employed and adapted the available bovine *ex vivo* platform to caprine PBMCs and used RNA-seq data to read out immune responses elicited by the highly virulent wild-type GM12 and several derivative mutants that have been constructed using synthetic genomics techniques and tested *in vivo* (except the mCherry-marked mutant) using the natural host. These mutants were GM12::YCpMmyc1.1 (virulent mutant of GM12 and parental strain of the other mutants used in this work) [21], its CPS-deficient mutant GM12::YCpMmyc1.1-Δ*glf* (attenuated *in vivo*) [17], GM12::YCpMmyc1.1-mCherry and GM12::YCpMmyc1.1-Δ68 (encapsulated but fully attenuated *in vivo*) [5]. This work confirms the switching of CPS surface expression in *Mycoplasma mycoides* subsp. *capri* [22]. By using an engineered capsule-deficient mutant, we were able to reveal in an unprecedented way the immunological consequences of the presence *versus* absence of CPS using a number of immunological assays.

## Results

### Capsular polysaccharide expression can switch in *Mycoplasma mycoides* subsp. *capri*

**Fig 1:**
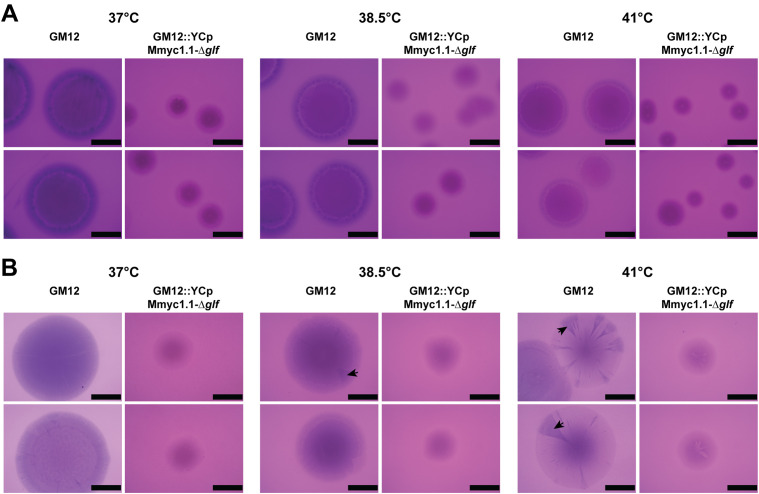
Effect of different growth conditions on *Mycoplasma* colony size and presence of polysaccharide. After four days of incubation the colonies were transferred onto a nitrocellulose membrane and subsequently stained using Periodic Acid-Schiff (PAS). Colony sectors stained for polysaccharide are indicated by black arrows. Scale bare is 1 mm. **(A)** Mycoplasmas were incubated at different temperatures on high nutrient agar. **(B)** Mycoplasmas were incubated at different temperatures on low nutrient agar.

First, we investigated whether CPS surface expression can vary in *Mycoplasma mycoides* subsp. *capri* and contribute to phenotypic diversity. Therefore, we grew *M. mycoides* subsp. *capri* strain GM12 (GM12) under different culture conditions and stained the colonies to observe CPS expression. Culture conditions tested included elevated temperatures that reflect fever-like conditions and different nutrient media compositions. First, we assessed phenotypic changes in *Mycoplasma* colonies by culturing GM12 on a high- or low-nutrient media and incubating it at different temperatures: 37°C, 38.5°C and 41°C. Periodic Acid-Schiff (PAS) staining confirmed the absence of CPS in individual sectors of the colonies grown at 41°C and starvation conditions, suggesting a temperature and nutrient-associated capsule on/off switch (Fig 1). The observed correlation of fever-like temperature and reduced detection of CPS made us employ a qRT-PCR assay targeting the UDP-galactopyranose mutase-encoding gene *glf* [16] involved in synthesis of galactofuranose. The downregulation of *glf* gene observed for GM12 cultured in low nutrient media or fever-like temperature correlate well with reduced detection of CPS under these conditions (S1 Fig).

### Doubling times of mycoplasmas used in this study

We investigated the doubling time of GM12 and its isogenic mutants (GM12::YCpMmyc1.1, GM12::YCpMmyc1.1-Δ68, and GM12::YCpMmyc1.1-Δ*glf*) in SP5 medium at 38.5°C. At 38.5°C, doubling times of GM12, GM12::YCpMmyc1.1, GM12::YCpMmyc1.1-Δ68 and GM12::YCpMmyc1.1-Δ*glf* were 66±4.4 min, 65±5 min, 73±6.3 min, and 63±5.5 min, respectively (S2 Fig). In conclusion, these results showed that all *Mycoplasma* strains included in the study expanded in a comparable manner at physiological body temperature.

### Impact of the capsular polysaccharide on interactions with caprine monocyte-derived macrophages (MDMs)

**Fig 2:**
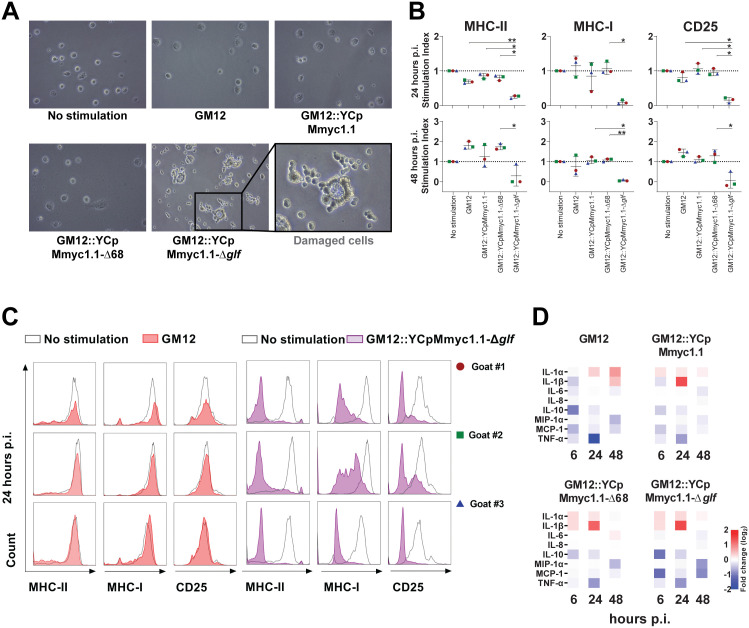
Cell morphology and activation/maturation of caprine monocytes-derived macrophages (MDMs) after stimulation with live mycoplasmas. (**A)** The MDMs (2 × 10^5^/mL in 12-well plate) were stimulated with mycoplasmas for 24 hours and their cell morphology was investigated by microscopy. Experiments were done in three biological replicates and representative data are displayed. Magnification of the pictures was 20x. (**B)** MDMs surface marker expression in response to stimulation with live mycoplasmas at 24 hours (upper panel) and 48 hours (lower panel), analyzed by flow cytometry. (**C)** Histogram of relative cell count versus activation markers (MHC-II, MHC-I and CD25) for MDMs stimulated with GM12 and GM12::YCpMmyc1.1-Δ*glf* compared to no stimulation. (**D)** Cytokines and chemokines secreted at 6, 24, and 48 hours after exposure to mycoplasmas, measured by multiplex immunoassay.

Since macrophages are involved in the primary immune response against bacterial infections in the respiratory tract, we generated MDMs to investigate their specific interactions with GM12 and its isogenic mutants. The results clearly indicated that only GM12::YCpMmyc1.1-Δ*glf* caused damage to MDMs, as witnessed by the extensive cellular monolayer disruption, whereas the other strains tested maintained the cell integrity comparable to unstimulated MDMs (Fig 2A).

We next tested whether those alterations of cell morphology could be related to altered MDM function/capacity to activate/maturate, using multi-parameter flow cytometry (FCM) assay. And indeed GM12::YCpMmyc1.1-Δ*glf* had a distinctive effect, by strikingly reducing the expression of MHC-II, MHC-I and CD25 at 24 hours post infection (p.i.); this reduction persisted up to 48 hours p.i. (Fig 2B and 2C). In contrast, GM12, GM12::YCpMmyc1.1, and GM12::YCpMmyc1.1-Δ68 had only moderate effects on MHC-II downregulation at 24 hours p.i.. However, the main effect observed for those three strains was a subsequent MHC-II upregulation at 48 hours p.i., as illustrated in Fig 2B. Moreover, by investigating cytokine secretion, we did not observe compelling differences in the cytokine responses to the strains investigated (Fig 2D).

These results inform that the capsule-deficient mutant GM12::YCpMmyc1.1-Δ*glf* mediates immunosuppressive (MHC and CD25 downregulation) as well as pro-inflammatory responses on macrophages *in vitro*, while the other encapsulated strains maintain prolonged interactions with host immune cells and upregulate MHC-II expression in MDMs up to 48 hours p.i..

### Capacity of GM12 to survive in caprine monocyte-derived macrophages (MDMs)

**Fig 3:**
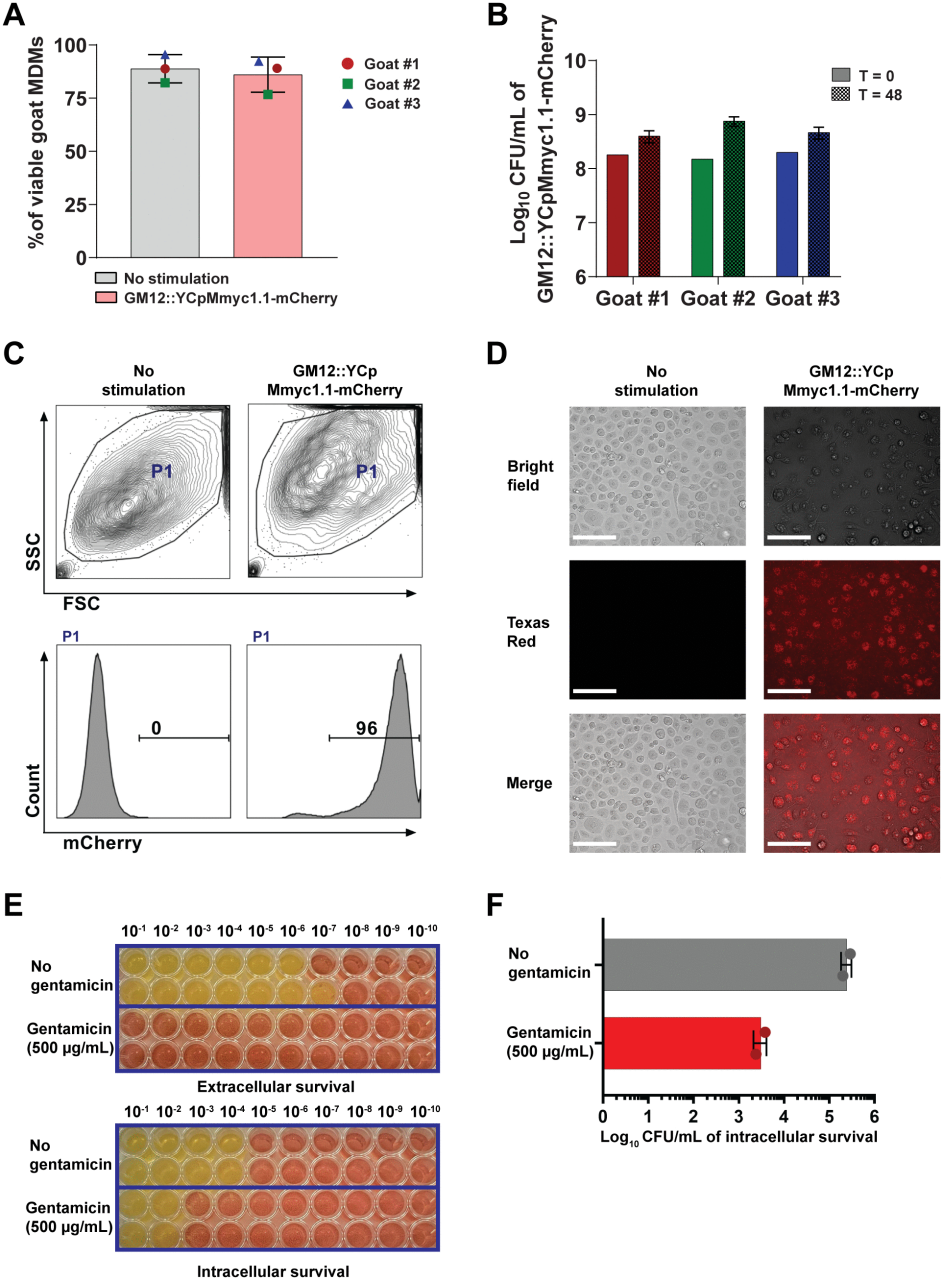
Viability of caprine monocyte-derived macrophages (MDMs) and *Mycoplasma mycoides* subsp. *capri* GM12 in co-culture. **(A)** Percentage of viable MDMs 48 hours post-infection with GM12 at an MOI of 100 was analyzed using the LIVE/DEAD Fixable Yellow Dead Cell Stain Kit. **(B)** Viability of mycoplasmas following co-culture with MDMs was determined by measurement of colony-forming units (CFUs) at 48 hours post-infection. **(C)** Flow cytometry analysis of phagocytosis after 48 hours of coincubation with mCherry-labeled GM12::YCp1.1. **(D)** Fluorescence microscopy images of infected MDMs 48 hours post-infection. Scale bare is 125 μm. **(E)** Viability of mycoplasmas following co-culture with MDMs determined by comparing color-changing units (CCUs) with or without treatment employing 500 μg/mL gentamicin (control = untreated). **(F)** The CFUs of intracellular mycoplasmas released from MDMs (H_2_O treatment) 48 hours post-infection.

To investigate survival in MDMs, we utilized an engineered mCherry-expressing strain, namely GM12::YCpMmyc1.1-mCherry. First, the expression of mCherry under the control of the spiralin promoter was confirmed using fluorescence microscopy and FCM analysis. This strain enabled us to investigate the potential binding to MDMs by microscopy and FCM assay. Result confirmed that GM12::YCpMmyc1.1-mCherry did not alter MDM viability (Fig 3A), while proliferating in the cell culture medium to 10^8^ CFU/mL at 48 hours p.i. (~ 1-log increase in all MDM samples derived from three independent animals) (Fig 3B). This indicated the inability of macrophages to kill all mycoplasmas that have been used for the infection in this specific assay format. Furthermore, when using fluorescence microscopy and FCM, we showed that over 95% of MDMs had attached or engulfed GM12::YCpMmyc1.1-mCherry, as evidenced by the high fluorescence intensity compared to unstimulated MDMs (Fig 3C and 3D).

However, at this stage it remained unclear how many GM12::YCpMmyc1.1-mCherry adhered mainly at the cell surface or within MDMs. To elucidate this, a classical gentamicin protection assay was performed with the parent GM12 strain. As seen in Fig 3E upper panel, 500 μg/mL gentamicin completely killed extracellular mycoplasmas after treatment (no colour change in all dilutions) in contrast to “no gentamicin” condition at 48 hours (colour change to yellow up to 10^-7^ dilution). Moreover, when we collected the MDMs (to assess GM12 “Intracellular survival”) and released their content by membrane disruption via a H_2_O treatment, we found a positive number of viable GM12 within MDMs, still ~2 × 10^3^ CFU/mL after gentamicin treatment, as illustrated in Fig 3E lower panel. This was confirmed by the CFU/mL enumeration (Fig 3F). The intracellular survival rate of GM12 within MDMs was low and around 0.012-0.019%. Moreover, confocal microscopy displayed extranuclear DAPI-stained spots within MDMs infected with mycoplasmas at 6 and 24 hours p.i., whereas those spots were absent without stimulation, as illustrated in S3 Fig. This extranuclear DAPI-stained spot pattern was previously reported to correspond to internalized mycoplasmas [23,24]. Interestingly, we could not find any co-localization between late endosome (Rab7, green) with mycoplasma’s putative DNA (DAPI, blue).

### Absence of capsular polysaccharide in GM12 affects viability of primary peripheral blood mononuclear cells (PBMCs)

Based on observations with MDMs, we next investigated the effect of live or heat-inactivated GM12 including its isogenic mutant on the viability of freshly isolated caprine or bovine PBMCs. Bovine PBMCs were included, since *Mmc* has been reported to be isolated from cattle [25] and we wanted to see whether PBMC viability depends on the host species used. Therefore, we quantified the percentage of dead cells after incubation with the mycoplasmas by utilizing LIVE/DEAD staining. We found that live GM12, GM12::YCpMmyc1.1 and GM12::YCpMmyc1.1-Δ68, and all heat-inactivated mycoplasmas did not affect viability of caprine or bovine PBMCs. In contrast, live GM12::YCpMmyc1.1-Δ*glf* strongly decreased caprine or bovine PBMC viability (S4A and S4B Fig, respectively). Specifically, viabilities of caprine and bovine PBMCs exposed to GM12, GM12::YCpMmyc1.1 and GM12::YCpMmyc1.1-Δ68 (live or heat-inactivated) remained above 70% and were comparable to the negative control. In contrast, stimulation with live GM12::YCpMmyc1.1-Δ*glf* led to a significant drop of viability to around 50%. As shown in S4C and S4D Fig, this increase of cell death was partially due to late apoptosis or necroptosis mechanisms. Altogether, the data suggests that the capsule-deficient mutant GM12::YCpMmyc1.1-Δ*glf* exposed their surface proteome including potential virulence traits such as lipoproteins, which likely induced necroptosis of PBMCs.

### Absence of capsular polysaccharide in GM12 triggers MHC-II downregulation in primary blood cells

**Fig 4:**
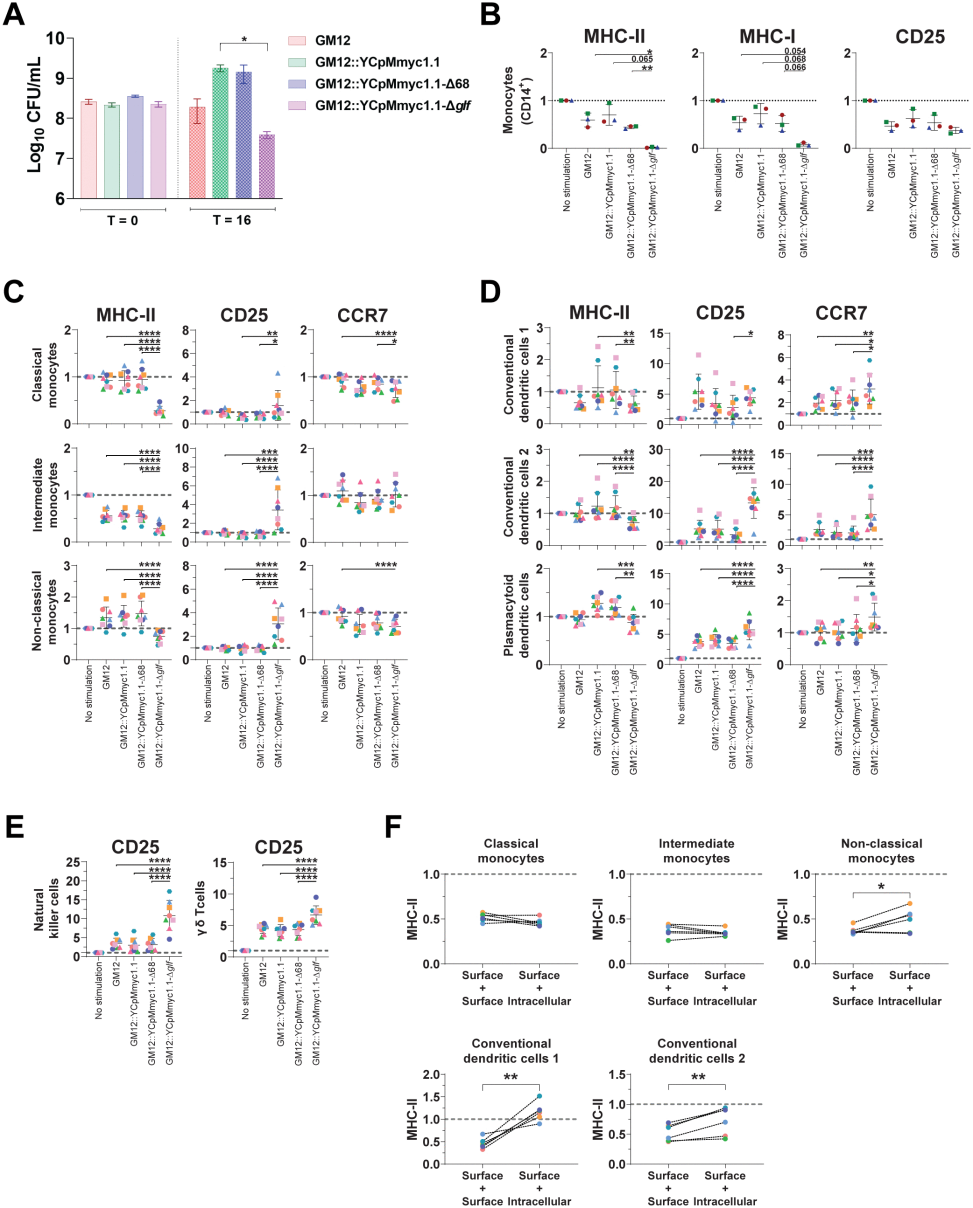
The absence of capsular polysaccharide impairs antigen presentation. **(A)** Viability of mycoplasmas following co-culture with caprine peripheral blood mononuclear cells (PBMCs) by comparing colony-forming units (CFUs) at T = 0 and T = 16 hours. A star indicates statistically significance by paired t-test. **(B–E)** Modulation of activation markers on (**B**) caprine monocytes (MHC-I, MHC-II, CD25), (**C**) bovine monocytes, (**D**) dendritic cells (MHC-II, CD25, CCR7), (**E**) natural killer (NK) cells and γδ T cells (CD25). Cells were exposed to mycoplasmas at a MOI = 100 for 16 hours. **(F)** GM12::YCpMmyc1.1-Δ*glf* induced MHC-II downregulation is due to a retention in the intracellular compartment on cattle PBMCs. The fold changes analysis was determined by FCM with FlowJo. Cells from the individual animals are represented by different color codes. Data are presented as mean ± standard deviation (*p < 0.05, **p < 0.01, ***p < 0.001, and ****p < 0.0001).

First, we determined the ability of mycoplasmas to replicate in DMEM supplemented with 10% FBS, following exposure to caprine PBMCs by comparing colony-forming units (CFUs) at T = 0 and T = 16 hours p.i.. After 16 hours p.i., the CFU/mL of GM12 remained constant, whereas GM12::YCpMmyc1.1 and GM12::YCpMmyc1.1-Δ68 showed a 1 log unit increase. Conversely, GM12::YCpMmyc1.1-Δ*glf* count decreased by around 1 log unit compared to pre-incubation (T = 0), as illustrated in Fig 4A.

Since the host innate immune response has been reported to play a crucial role in the control of many bacterial infections, we then focussed our attention on antigen presenting cells (APCs), essential for bridging innate and adaptive immune systems, and thus triggering the T cell response. To characterize the response of caprine APCs to mycoplasmas, a multi-parameter FCM assay was applied to analyse MHC-II, MHC-I, and CD25 expression levels on CD14^+^ monocytes. Again, GM12::YCpMmyc1.1-Δ*glf* had distinctive effect compared to other strains tested, this time by clearly down-regulating the expression of MHC-II and MHC-I. However, no statistically significant differences were observed in CD25 expression for all tested mycoplasmas (Fig 4B). Importantly, this GM12::YCpMmyc1.1-Δ*glf* induced MHC-II downregulation on CD14^+^ cells was unrelated to cell death engagement, since this observation was found on Annexin V^-^/ 7-AAD^-^, Annexin V^+^/ 7-AAD^-^ and Annexin V^+^/ 7-AAD^+^ monocytes (S4E Fig).

Although goats are the native host species for *Mmc*, the immune system of this ruminant species has been less studied, with a void of specific or cross-reactive antibodies to delineate caprine immune cell subsets. This prompted us to transfer our experimental approach to bovine immune cells, where a more developed range of tools is even commercially available (S5 Fig). Moreover, it was reported that cattle can get infected with *Mmc* as well [25] as already mentioned above. Indeed, by doing so, we could not only include additional activation/maturation markers (CCR7), but we could also investigate other highly specialized APC subsets in addition to monocytes, namely dendritic cells (DCs). Confirming previous observations in goat, the results show that GM12::YCpMmyc1.1-Δ*glf* suppressed the expression of MHC-II on both monocytes and DCs, the latter being particularly efficient for antigen presentation. Interestingly, downregulation of activation/maturation marker was not systematic, since in the meantime GM12::YCpMmyc1.1-Δ*glf* increased the expression of CD25 (monocyte and DC subsets) and CCR7 (dendritic cells) (Fig 4C and 4D). Altogether, these results showed the remarkable capacity of engineered CPS-negative *Mmc* to differentially modulate ruminant immune cells.

Another advantage to work with bovine immune cells was the possibility to investigate other subsets bridging innate-adaptive immunity (such as natural killer cells (NK cells) and γδ T cells). Effectively, we expanded our findings by showing that GM12::YCpMmyc1.1-Δ*glf* increased the expression of CD25 on both immune cell subsets, as shown in Fig 4E.

A possible explanation for the detected MHC-II downregulation would be the inaccessibility of freshly generated MHC-II molecules to the plasma membrane. To test this hypothesis, we compared between FCM staining procedure “cell surface only” and “cell surface followed by intracellular staining”, the latter cumulating detection of MHC-II molecules already processed at the plasma membrane and cytosolic MHC-II molecules yet to be transported at the cell surface. Clearly, the previously observed MHC-II downregulation in response to GM12::YCpMmyc1.1-Δ*glf* no more persisted when intracellular compartment could be reached, indicating that MHC-II molecules encountered a cellular relocation rather than a decrease in numbers. This MHC-II molecules retention within the intracellular compartment, particularly in professional APCs (cDC1 and cDC2) (Fig 4F), was likely to have a negative outcome on the subsequent immune response.

Having focussed our efforts on the host side, we then considered how *Mmc* colonies could be impacted following physical contact with caprine blood cells, notably to explain why GM12::YCpMmyc1.1-Δ*glf* titers decreased following exposure to PBMCs (S6 Fig). For adhesion assessment, caprine PBMCs were added on the top of GM12 and GM12::YCpMmyc1.1-Δ*glf* colonies for 30 minutes. While adhesion results proved to be comparable between GM12 and capsule-deficient mutant (same density of blood cells sticking to bacterial colonies observed by inverted microscopy), colonies of the capsule-deficient mutant were notably fragile compared to GM12 counterparts, as witnessed by the decrease in size and colony edge disruption. This observed instability might contribute to the aforementioned decrease of GM12::YCpMmyc1.1-Δ*glf* titers in the supernatant of exposed PBMCs for 16 hours (Fig 4A).

### Capsular polysaccharide prevents a pro-Th1 cytokine response

**Fig 5:**
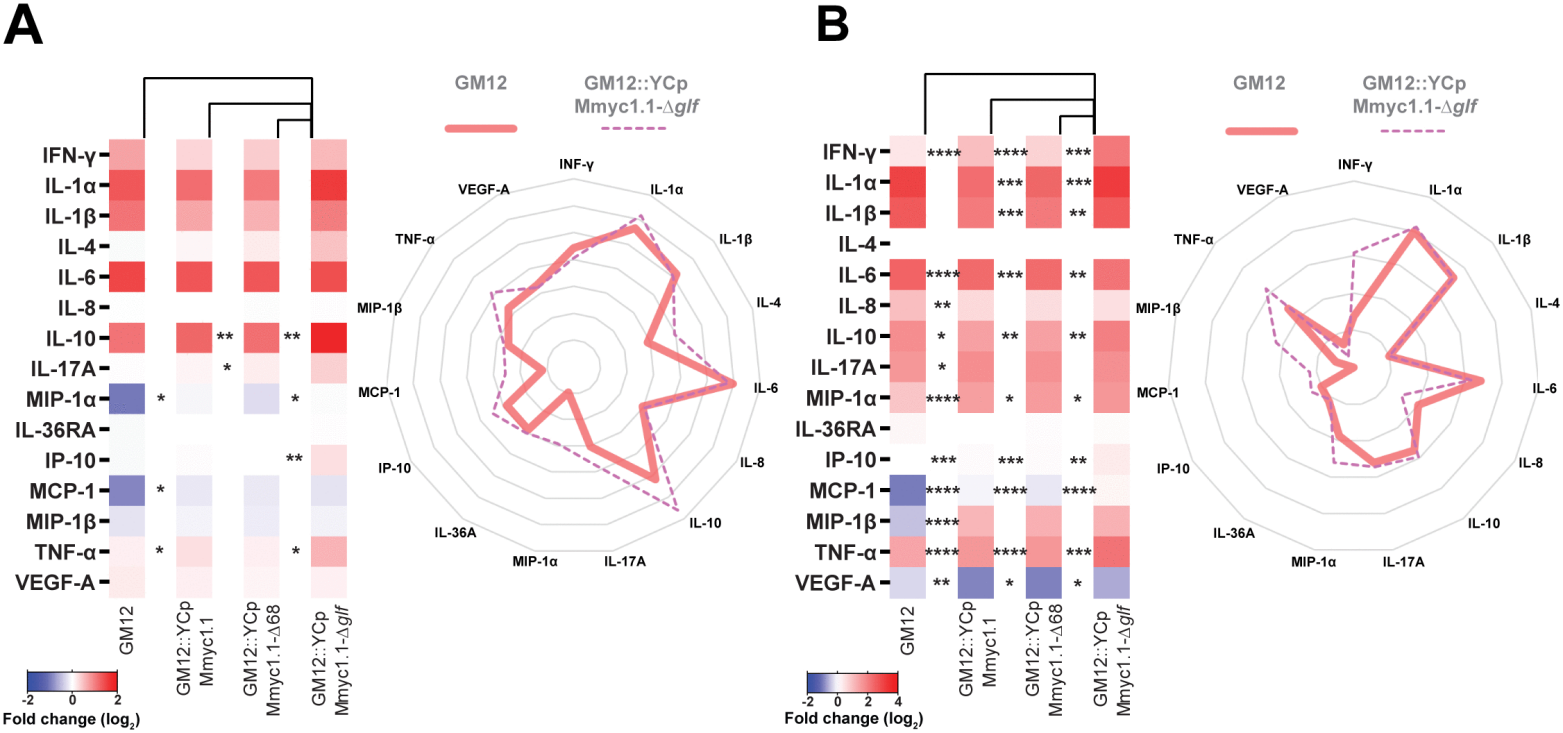
Cytokines and chemokines secreted by peripheral blood mononuclear cells (PBMCs) stimulated with mycoplasmas. Supernatants were harvested 16 hours post infection and measured by a commercial multiplex immunoassay. **(A)** Cytokines/chemokines secreted by caprine PBMCs, (**B**) cytokines/chemokines secreted by bovine PBMCs. Stars indicate significance levels (*p < 0.05, **p < 0.01, ***p < 0.001, and ****p < 0.0001).

The host defense mechanisms against infections with pathogenic mycoplasmas involves sequentially components of innate and adaptive immune responses, the latter requiring the production of Th1, Th2 and Th17 cytokines. We employed a multiplex immunoassay to determine the levels of secreted cytokines produced by caprine PBMCs exposed to mycoplasmas. Due to the lack of a dedicated caprine-specific assay, we used a bovine assay (MILLIPLEX Bovine Cytokine/Chemokine Magnetic Bead Panel 1) capable of measuring caprine cytokines (except IL-8) thanks to cross-reactivity (Fig 5A, heatmap summarizing fold-inductions; S1 Table, p value of GM12::YCpMmyc1.1-Δ*glf* compared to other strains; S7A Fig, Log concentrations of all individual animals). The results indicate that all tested strains mediated pro-inflammatory cytokines secretion by caprine PBMCs. Importantly, bovine PBMCs exhibited similar trends, as illustrated in Fig 5B (Log_2_fold change), S2 Table and S7B Fig (Log concentrations), showing again that cattle can be used as alternative model to goats for an in-depth study of host-*Mmc* interactions.

The levels of the Th1 cytokines (IFN-γ), as well as pro-inflammatory (TNF-α, MIP-1α, IP-10) and anti-inflammatory cytokines (IL-10) were significantly elevated following stimulation by GM12::YCpMmyc1.1-Δ*glf* compared to other *Mycoplasma* strains. Other cytokines, such as IL-1α, IL-1β, IL-8, IL-17A, and IL-36RA were only marginally modulated. Cytokines IL-4 and VEGF-A were below the detection threshold in all samples, likely due to either their low abundance or absence of secretion in primary blood cells under the tested conditions (S7A and S7B Fig). These results indicated that primary blood cells stimulated with all mycoplasmas strains secreted a bulk of pro-inflammatory cytokines that likely promote a robust and balanced T-cell mediated response, with the most potent effect (particularly for IFN-γ and TNF-α) observed for the capsule-deficient mutant GM12::YCpMmyc1.1-Δ*glf*.

### Intracellular cytokines were mainly produced by pDCs and NK cells in response to capsule-deficient mutant

**Fig 6:**
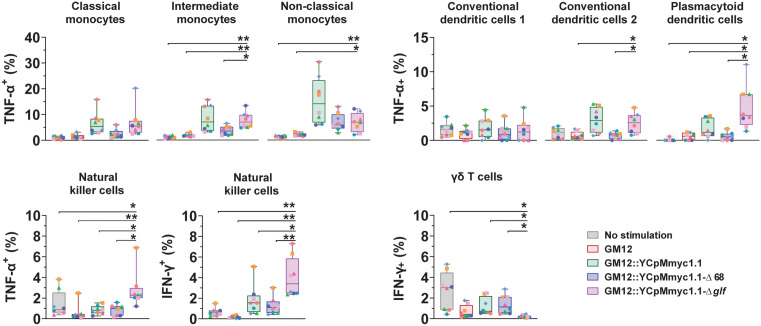
Intracellular cytokines produced by bovine plasmacytoid dendritic cells (pDCs) and natural killer (NK) cells in response to GM12::YCpMmyc1.1-Δ*glf.* Intracellular cytokine staining of TNF-α and IFN-γ. Following stimulation with mycoplasmas for 16 hours, intracellular TNF-α and IFN-γ were determined by flow cytometry. Stars indicate significance levels (*p < 0.05 and **p < 0.01).

As reported in our previous study, four antibodies were employed to detect intracellular cytokines by FCM, namely IFN-γ, TNF-α, IL-4 and IL-17. Bovine PBMCs did not produce detectable levels of IL-4 and IL-17 following *Mmc* exposure. GM12::YCpMmyc1.1 and GM12::YCpMmyc1.1-Δ*glf* were exclusively able to trigger TNF-α secretion by DCs and monocytes, both to a comparable level. The only cell subset for which GM12::YCpMmyc1.1-Δ*glf* further increased TNF-α secretion compared to GM12::YCpMmyc1.1 were NK cells. From this, it appeared unclear whether the absence of CPS or presence of the YCp element in the genome triggered the production of TNF-α. However, the results were far more convincing related to IFN-γ, where particularly elevated amounts of this pro-Th1 cytokine were seen for NK cells, confirming their response towards mycoplasmas. In contrast, the other cell subsets contributed weakly to IFN-γ production, as illustrated in Fig 6.

### The capsule-deficient and GM12 wild-type strains induced distinct transcriptomic profiles in caprine PBMCs

To gain better understanding of host-pathogen interactions at the cellular response level, caprine PBMCs were infected with mycoplasmas at MOI = 100 and their transcriptome profiles were determined at 6 and 16 hours p.i.. The 6-hour time point was included to monitor early innate immune responses that begin as soon as four hours after exposure to infectious agents. Since transcriptomic analyses do not face similar limitations as in phenotypic investigation, this part of the study was done with caprine cells only.

**Fig 7:**
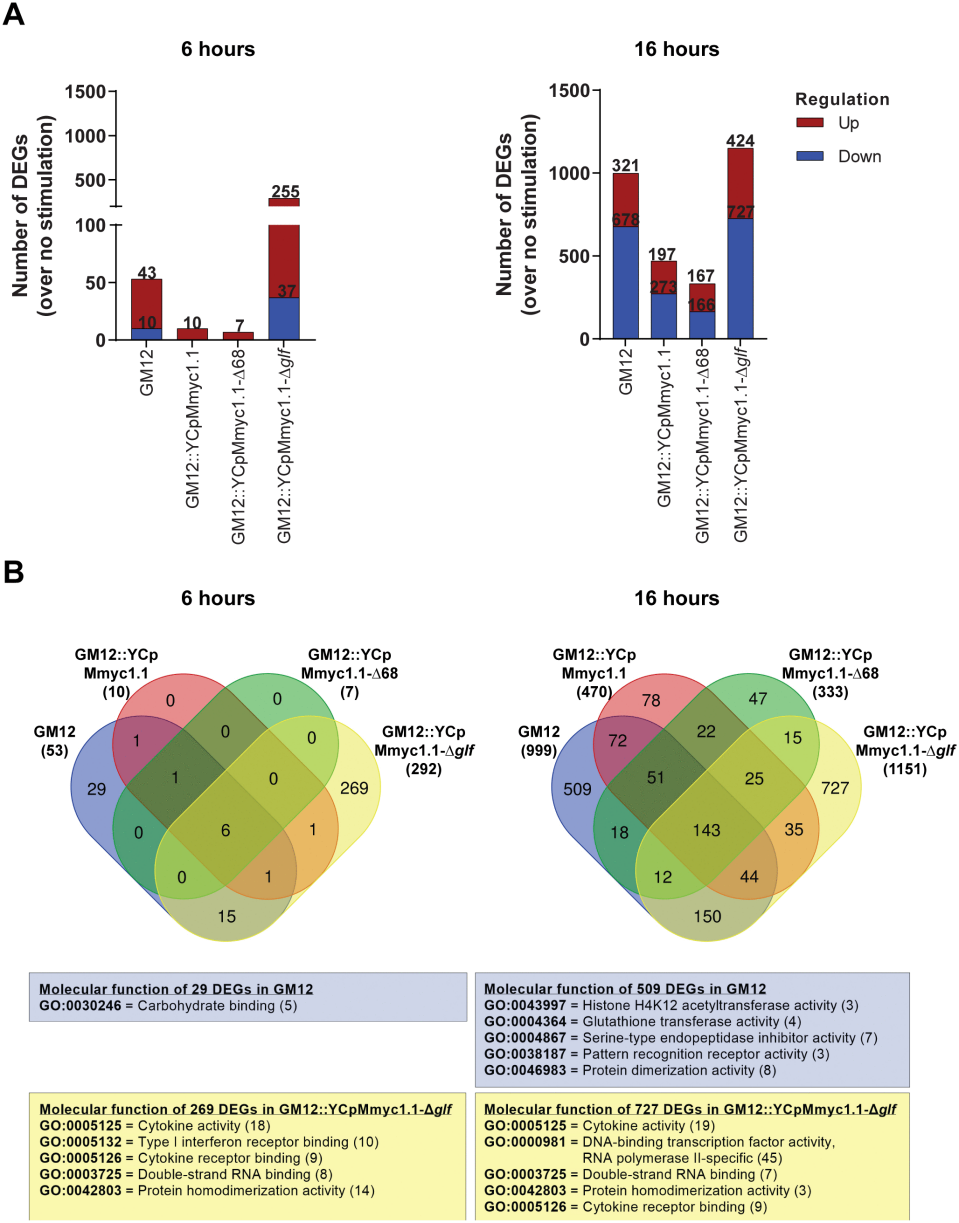
Number of differentially expressed genes (DEGs) in caprine peripheral blood mononuclear cells (PBMCs) stimulated with mycoplasmas for 6 hours (left panel) and 16 hours (right panel). **(A)** Number of DEGs in infected PBMCs over no stimulation at 6 hours and 16 hours. **(B)** Venn diagram of DEGs among four comparisons (GM12 vs. no stimulation, GM12::YCpMmyc1.1 vs. no stimulation, GM12::YCpMmyc1.1-Δ68 vs. no stimulation and GM12::YCpMmyc1.1-Δ*glf* vs. no stimulation). The number in each large circle represents the total number of DEGs between combinations, the overlap part of the circles represents common DEGs. DEGs were annotated on the basis of gene ontology (GO) using DAVID (Database for Annotation, Visualization and Integrated Discovery).

Differentially expressed genes (DEGs) were compared in PBMCs stimulated with different mycoplasmas versus unstimulated cells. At 6 hours p.i., we found that only GM12 and GM12::YCpMmyc1.1-Δ*glf* had clear effects on PBMCs by increasing the number of DEGs; 53 and 292, respectively. In contrast, exposure to GM12::YCpMmyc1.1 and GM12::YCpMmyc1.1-Δ68 led to the induction of only a limited number of DEGs (Fig 7A left panel). At 16 hours, the number of DEGs were increased for all mycoplasmas tested, but GM12 and GM12::YCpMmyc1.1-Δ*glf* triggered the induction of the highest number of DEGs: 999 and 1151, respectively (Fig 7A right panel). A Venn diagram summarizing the results showed that 29 and 509 DEGs were found to be unique to GM12 infection at 6 and 16 hours, respectively. Importantly, the upregulated genes in GM12-stimulated PBMC are involved in pathogen recognition, initially and more specifically in carbohydrate binding (Fig 7B left panel). However, this appeared to be moderate compared to PBMCs stimulated by GM12::YCpMmyc1.1-Δ*glf*, where 269 DEGs at 6 hours and 727 DEGs at 16 hours were identified exclusively in response to this specific strain. These DEGs confirmed the broad immunostimulation profile described above with our *ex vivo* platform, with a particular trend for inflammatory responses (Fig 7B right panel).

### The capsule-deficient mutant induces an antiviral activity-like transcriptome associated with a robust inflammatory response in caprine PBMCs

**Fig 8:**
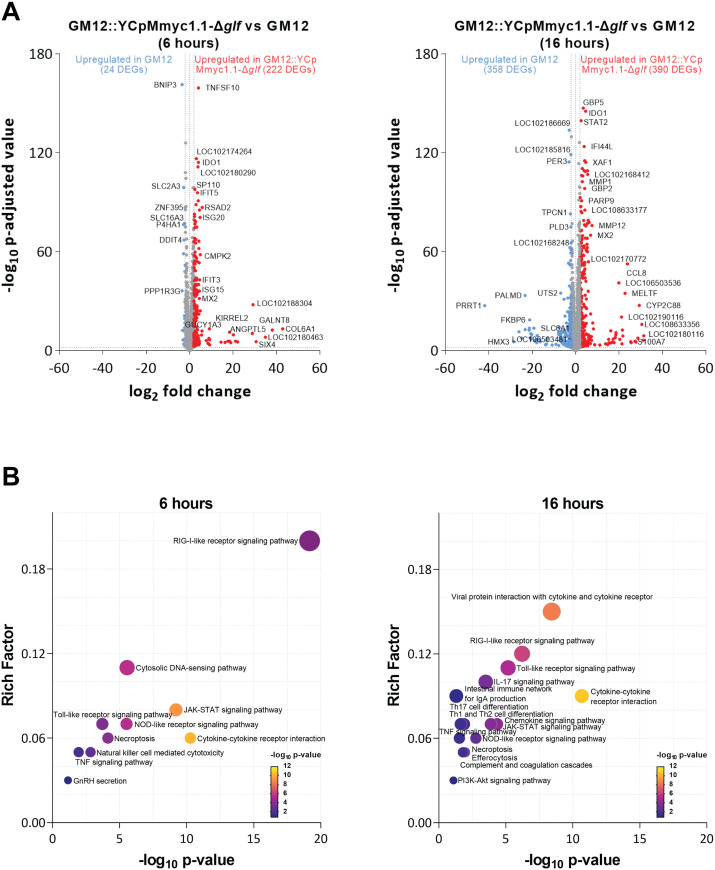
Number of differentially expressed genes (DEGs) in caprine peripheral blood mononuclear cells (PBMCs) stimulated with mycoplasmas for 6 hours (left panel) and 16 hours (right panel). **(A)** Volcano plot and **(B)** KEGG pathway of 246 DEGs at 6 hours and 748 DEGs at 16 hours compared between GM12::YCpMmyc1.1-Δ*glf* and GM12. The line represents the significant genes at adjusted p value of < 0.05 and absolute log_2_ fold change > 2.

We then aimed to provide mechanistic insights in PBMCs stimulated by GM12 and GM12::YCpMmyc1.1-Δ*glf.* We used Volcano plots that highlighted the most significantly expressed transcripts: 246 DEGs at 6 hours (Fig 8A left panel and S3 Table) and 748 DEGs at 16 hours (Fig 8A right panel and S4 Table) in GM12::YCpMmyc1.1-Δ*glf* over GM12 infected cells. Remarkably, both strains led to very distinct transcriptomic signatures at 6 and 16 hours, confirming that the absence of CPS alters drastically the host-pathogen interaction, inducing differential expression of immune related genes.

To gain insights into the biological functions of DEGs, genes that were significantly differentially expressed between GM12 and GM12::YCpMmyc1.1-Δ*glf* were analyzed using DAVID (Database for Annotation, Visualization and Integrated Discovery) based on KEGG pathways and gene ontology (GO) terms. At 6 hours, 222 up-regulated genes (mainly in GM12::YCpMmyc1.1-Δ*glf*) encoding proteins reported for antiviral responses, including RIG-I-like receptor pathway (17 genes), cytosolic DNA-sensing pathway (10 genes), and cytokine-cytokine receptor interaction (18 genes), while 24 down-regulated genes (mainly in GM12) were linked to GnRH secretion as shown in Fig 8B and S5 Table. At 16 hours, 390 up-regulated genes were involved in similar pathways as those observed at 6 hours, with additional involvement in adaptive immune responses, such as IL-17 signalling pathway, Th17 cell differentiation, Th1 and Th2 cell differentiation (Fig 8B and S6 Table). Confirming this, the GO analysis is depicted in the S8A and S8B Fig. Interestingly, the capsule-deficient mutant selectively up-regulated *RIPK3* and *MLKL* genes compared to GM12 (S9 Fig), consistent with necroptosis as shown in KEGG pathway at both time points (Fig 8B). These findings indicate that the capsule-deficient mutant triggers an inflammatory cell-death program such as necroptosis, rather than immunologically silent apoptosis. In conclusion and as expected from results described above, our transcriptomic analysis revealed that the absence of CPS had major impacts on the transcriptome of caprine PBMCs, notably by up-regulating genes involved in antiviral responses, inflammation, and cell death. These findings align well with the phenotypic effects observed in both MDMs and PBMCs.

## Discussion

*Mycoplasma mycoides* is a ruminant pathogen of utmost importance in Africa affecting not only the health and productivity of ruminant livestock but also the livelihoods of thousands of livestock dependent people. The biosafety level 3 (BSL-3) security requirements for *Mycoplasma mycoides* subsp. *mycoides* (*Mmm*) in many countries [26] and the inability to mutagenize its genome by state-of-the-art synthetic genomics tools [27] favor *Mycoplasma mycoides* subsp. *capri* strain GM12 (*Mmc* GM12) as a model organism to decipher host-pathogen interactions for the species, since a well-established infection model is available [5] and the genome can be modified using synthetic genomics tools [15]. The use of cells and tissues of the native host species is relevant for getting a meaningful insight into host-pathogen interactions, especially for mycoplasmas that are known for a high degree of host- and tissue specificity. We used ruminant primary immune cells to decipher interactions with *Mmc* GM12, a highly virulent strain that has been shown to cause septicemia after experimental challenge *in vivo* [5,17,21].

First, we aimed to investigate whether CPS can contribute to phenotypic diversity of different *Mmc* strains. Therefore, we investigated colonies grown under different culture conditions, and observed distinct colony segments that did not stain against CPS (S10 Fig). Our finding is very much in line with data previously reported for *Mmm* colonies that also vary their CPS surface expression [12,28,29] and its inflammatory properties as well *in vitro* and *in vivo*. The availability of host’s glucose, the main building block of CPS is likely to vary in different tissues, such as the mucosal airway with low glucose concentration in its mucus [30], which might also effect the CPS surface expression, but was not tested in this study due to the complex media requirements for axenic growth of mycoplasmas. A number of gene families in mycoplasmas are affected by frequent, stochastic genotypic changes, events that are able to alter the expression of surface molecules [31], which might also apply for the CPS.

Our basic hypothesis upon initiation of this study was that in the absence of known toxins, host-*Mmc* interactions drive inflammation which is a hallmark of disease. Most likely specific immune cell subsets drive inflammation, which we aimed to narrow down. For the study, we used a number of engineered *Mmc* mutants including one incapable to produce galactofuranose (capsule-deficient mutant) [17] and another one that is lacking 68 genes and was fully attenuated *in vivo* [5].

Our data point to an effect of the yeast centromeric element (YCp) in terms of phenotypic fitness, since we observed at times differences between the strain GM12 and the derivative mutant GM12:YCpMmyc1.1. Differences were already indicated in previous *in vivo* challenge trials, where GM12::YCpMmyc1.1 [17,21] was slightly less virulent than its parental wild type strain GM12 [5]. It is likely that a minimal organism is easily affected by additional genetic elements and future studies on host-pathogen interactions would benefit from marker-free mutants.

However, the lack of immune biologicals to characterize the caprine immune responses restricted the in-depth phenotypic investigation. We thus complemented the caprine studies with a number of assays based on bovine cells, which are also relevant since *Mmc* has also been isolated from diseased cattle [25]. The use of appropriate experimental models is crucial for understanding pathogen tropism like in the case of mycoplasmas, which are hosts and tissue specific. Basically, we applied a recently developed *ex vivo* platform that encompasses most circulating immune cell subsets in animal species [18]. Of note, since the latter study emphasized to perform immunological assays at the host’s body temperature, we verified differences of assay outcomes at 37°C (“standard cell culture conditions, ruminant hypothermia”) and 38.5°C (ruminant body temperature) (S11 Fig), and opted to perform all tests at 38.5°C. Consequently, our results might deviate from data collected at 37°C.

One might argue a discrepancy between the importance of a capsule and what was previously reported *in vivo*, where the encapsulated GM12::YCpMmyc1.1-Δ68 showed full attenuation *in vivo*, but the capsule-deficient mutant GM12::YCpMmyc1.1-Δ*glf* led to mild symptoms. A capsule whose synthesis relies on two dynamic genetic loci in *Mycoplasma mycoides* [22], is not necessarily the one and only virulence factor. In infectious diseases the adhesion and colonization mark the first and crucial step of pathogenesis. In fact, we have shown that the absence of a capsule at least *in vitro* promotes adhesion [16]. The encapsulated GM12::YCpMmyc1.1-Δ68 misses a large number of lipoprotein-encoding genes among other candidate virulence factor-encoding genes, which probably contribute to adhesion and colonization. Therefore, our results do not contradict the *in vivo* results obtained with GM12::YCpMmyc1.1-Δ68 and GM12::YCpMmyc1.1-Δ*glf*.

Ruminant mycoplasmas are generally considered extracellular pathogens, but a growing body of evidence suggests that several species, such as *Mycoplasmopsis agalactiae* and *M. bovis*, can adopt an intracellular lifestyle, allowing them to evade detection by both non-phagocytes and phagocytes [32,33] as well as avoiding humoral immune responses. Given that *M. mycoides* enters the host via the respiratory tract, we employed MDMs as proxy of recruited macrophages entering the respiratory tract during the early phase of infection [34]. Indeed, increased levels of macrophages have been identified *in situ* in lung tissues of CBPP-affected cattle [35], while macrophage interactions with *Mmm* have been recently investigated [36]. Herein, we have optimized the protocol according to up-to-date procedures [37], notably to overcome the low affinity of CD14 antibodies in goat, ending up with a pure fraction of positively-selected monocytes rather than the bulk of PBMC population, enhancing the consistency and reliability of our findings. In our study only the capsule-deficient mutant negatively impacted the viability of MDMs as well as their capacity to express MHC-I, MHC-II and CD25, while the mCherry-tagged GM12 survived in low numbers in macrophages. A survival of *Mmm* in bovine macrophages without preincubation with opsonizing antibodies was reported recently, which supports our findings [36]. Such survival would explain a possible mode of dissemination, which has been reported for a number of pathogenic mycoplasmas. According to our results, theoretically a cease in CPS surface expression inside macrophages would kill the macrophage and release the mycoplasmas into different body sites. With respect to the cytokine secretion of the MDMs, we observed the release of IL-1, which matches published *in situ* data from CBPP-affected cattle [35]. Finally, although we have optimized monocyte sorting procedure and used well established differentiation into macrophage-like cells by GM-CSF, one must keep in mind that our system cannot recapitulate the nuance and plasticity of macrophage polarization during *in vivo* infection (macrophage lineages but also monocyte derived DC like cells, surpassing inflammatory events). Altogether, our findings recapitulate how *Mmc* GM12 can survive in macrophages and persist within the host and the pivotal role of CPS for this. This likely contributes to disease severity, emphasizing the need for further research to explore *Mmc*’s immune evasion strategies.

Next, we investigated the responses of PBMCs towards *Mmc*. Interestingly, the capsule-deficient mutant revealed the strongest pro-apoptotic effect on PBMCs (S4 Fig), in line with the findings related to the survival of macrophages. Exposed lipoproteins as well as other surface molecules are likely to be the main players responsible for this effect. Interestingly, a similar effect was evident, when the wild-type strain GM12 was grown in media with less nutrients at fever like temperatures (S12 Fig and S7 Table). CD14^+^ caprine monocytes had down-regulated expression of MHC-I and -II after exposure to GM12::YCpMmyc1.1-Δ*glf*, negatively impacting the bridge of innate and adaptive immune responses. When employing bovine PBMCs we also detected the downregulation of MHC-II in monocytes and dendritic cells with potential negative impacts on antigen presentation, showing for the first time that this immunosuppressive mechanism is directly associated with lack of CPS expression. Moreover, our study revealed that MHC-II downregulation at the surface of monocytes, dendritic cells and MDMs is largely attributed by cytosolic retention; nevertheless, the exact signaling pathways remains to be elucidated and could offer important insights into *Mollicutes* pathogenesis. The observed discrepancy of CD25 upregulation in monocytes and other immune cell subsets (Fig 4) *versus* downregulation in MDMs (Fig 2B and 2C) exposed to GM12::YCpMmyc1.1-Δ*glf*, is probably due to the presence (PBMCs) *versus* absence (MDMs) of T cells, the main source of IL-2. Next we investigated the cytokines secreted by PBMCs and we detected a number of cytokines previously measured in plasma [38] from cattle experimentally infected with *Mmm* [39]. Stimulation by GM12 led to a number of pro-inflammatory cytokines and the Th1 cytokine IFN-γ, while GM12::YCpMmyc1.1-Δ*glf* was most effective in inducing their secretion in contrast to the other strains tested. Intracellular cytokines were mainly produced by pDCs and NK cells. The transcriptomic profiles of PBMCs infected with GM12 and the CPS-deficient mutant differed substantially corroborating the phenotypic results reported here. Specifically, the CPS-deficient mutant GM12::YCpMmyc1.1-Δ*glf* strongly activated genes linked to antiviral defence and pro-inflammatory cytokines. Of note, the CPS-deficient mutant mediated antiviral gene expression (such as *IFN-ω*, *IFN-β*, *IRF7*, and *ISG15*) showed overlapping pathways between bacterial and viral responses; however, the precise mechanism, although providing potential important insights into the pathogenesis of *Mmc*, remains to be elucidated and was out of scope of the present study. Finally, one must consider the limitation of the present work, that did not provide transcriptomic profiles at later stages than 16 hours, which would have revealed additional insights into the host immune response triggered by *Mmc* over time.

Components of the pathogen’s surface have a crucial role in adhesion and colonization of the host. We were surprised that the mutant lacking 68 genes and being fully attenuated *in vivo* mirrored many results obtained by its parental strains. These data pinpoint to a loss of genes encoding factors for adhesion, colonization and longer-term survival, that were not investigated in our study. Indeed the mutant strain lacking 68 genes lacks the MIB-MIP system [10], the peroxide production system [5] and many lipoproteins that are key for adhesion and colonization [8].

In this study, we clearly demonstrate for the first time that the presence of CPS correlates with modest immune responses. Different roles of CPS for different mycoplasmas have been reported by the research community including bacterial adhesion to epithelial cells [40,41], downregulating alveolar macrophage responses [42], providing protection against complement-mediated lysis [12,43] and inducing inflammatory responses [44], which we partly confirmed in our study. We showed for the first time phenotypic diversity related to the presence or absence of CPS and its dramatic consequences on the immunological cascade induced by *Mmc*. Our data support a role of CPS in an immunological furtiveness state of *Mmc* that would explain a carrier status of animals as observed for many mycoplasmas [45]. A previous study tested a glycoconjugated vaccine candidate with a promising outcome, indicating that the capsule is a vaccine target for *Mycoplasma mycoides* [46].

Surface phenotypic diversity of *Mycoplasma mycoides* in terms of lipoproteins expressed and the presence or absence of CPS is likely to occur inside the host affecting the spectrum of immune responses. Therefore, different subsets of mycoplasmas are likely to occupy specific niches with respect to host’s tissues or cells supporting the survival of the pathogen in the hosts, according to the diversity-stability hypothesis. We were not able to determine the percentage of CPS-positive GM12 cells in our experiments, due to technical limitations. Nevertheless, the difference to the CPS-negative locked phenotype of GM12::YCpMmyc1.1-Δ*glf* was evident in many experiments suggesting a large proportion of CPS-positive cells among GM12, GM12::YCpMmyc1.1 and GM12::YCpMmyc1.1-Δ68. To summarize, the capacity of *Mmc* to modulate CPS at the surface induces diverse and antagonistic effects on immune host cells. Depending on the tissues targeted and the course of infection, this phenotype diversity based on CPS on/off switch at the surface, allows the binding and survival of *Mmc* in host immune cells, but also inhibition of induction of adaptive response, offering best conditions for persistence and dissemination.

## Conclusion

In this work we confirmed that *Mmc* can switch its expression of CPS, which increases the spectrum of phenotypic diversity of the pathogen in the host besides the already reported variation of surface lipoprotein expression [47,48]. The immunological consequences of the presence or absence of CPS are apparent and a strong immune response is likely to foster clearance which is supported by the *in vivo* confirmed data employing the CPS-deficient mutant. Therefore, CPS contributes to an immunological furtiveness like state in *Mycoplasma mycoides*. Immunological responses of the macrophages and PBMCs are not likely main drivers of host specificity but rather of inflammation.

## Materials and methods

### Ethics statement

The collection of ruminant blood was performed in compliance with the Swiss animal protection law (TSchG SR 455; TSchV SR 455.1; TVV SR 455.163). The application was reviewed by the Cantonal Committee on Animal Experimentation of the cantons of Bern, Fribourg and Solothurn. The veterinary authority of the canton of Bern (Amt für Landwirtschaft und Natur LANAT, Veterinärdienst VeD, Bern, Switzerland) approved the animal experiments under the licenses BE55/2022, BE127/2020 and BE104/2023.

### *Mycoplasma* strains and culture conditions

The mycoplasmas utilized in this study are listed in S8 Table. All strains were grown at 37°C or 38.5°C at 5% CO_2_ in SP5 medium (pH 7.5) [49]. Growth curves and doubling times were generated as recently described [50]. For immunological assays, mycoplasmas were harvested at the log phase, stored at -80°C until further use. Colony forming unit (CFU) per mL were determined from frozen aliquots.

To heat-inactivate mycoplasmas, an aliquot was thawed and heated at 65°C for 10 minutes before being employed for immunological assays. Heat-inactivation was confirmed by the absence of growth in liquid media.

### Characterization of capsular polysaccharide surface expression under different culture conditions

Mycoplasmas were inoculated in 5 mL of Hayflick medium (high- or low-nutrient) or SP5 medium (S9 Table), and incubated at 37°C, 38.5°C, or 41°C overnight. Cultures were then transferred at a 1:1,000 dilution into fresh media and incubated overnight at the respective temperatures. Overnight cultures were harvested for RNA extraction and serial dilutions were plated on agar plates and incubated for four days. Polysaccharide production was screened by colony blots. Therefore, we adapted the colony blotting and Periodic Acid-Schiff (PAS) staining assay using the Glycoprotein Detection kit (Sigma-Aldrich, USA). Colonies were transferred onto a nitrocellulose membrane (Merck Millipore, Ireland) by gently pressing the membrane onto the agar surface for 5 minutes. The membranes were then fixed in methanol (1:1 v/v) for 30 minutes at room temperature, followed by two washes in distilled water (10 minutes each). For PAS staining, membranes were incubated in oxidation solution for 30 minutes, washed twice with distilled water (10 minutes each), and then incubated in Schiff reagent for 2 hours. Finally, the membranes were treated with a reduction solution for 1 hour and washed twice in distilled water (10 minutes each). *Mycoplasma* phenotypes were then examined under a Nikon SMZ18 stereo microscope (Nikon, Japan).

### Construction of GM12::YCpMmyc1.1-mCherry

The mCherry expressing mutant was generated using TREC-IN [51] and all primers used are listed in S10 Table. Basically, the LacZ-encoding gene of the *Mmc* genome YCpMmyc1.1 [15,52] cloned in *Saccharomyces cerevisiae* strain VL6-48N [53] was replaced by the mCherry-encoding gene in three steps. Step 1: Insertion of the CORE6 cassette at the target locus via homologous recombination and selection for Uracil prototrophy; The CORE6 cassette contains 50 bp stretches homologous to 50 bp sequences flanking the gene to be removed, a I-SceI recognition site, the I-SceI gene under the control of the Gal1 promoter, and a non-functional truncated antibiotic resistance gene module (5’ kanMX). Step 2: Site-specific integration of a second construct containing the 3’ truncated kanMX gene component which, when integrated properly restores functionality of the antibiotic resistance (geneticin resistance). It also contains the gene of interest (mCherry) and a repeat of the 50 bp upstream of the gene to be removed. Step 3: Removal of the CORE6 cassette via galactose induction of the I-SceI endonuclease, producing a double-strand break at the I-SceI recognition site, and enhancement of homologous recombination between the two 50 bp repeat sequences present in the CORE6 and in the second construct 5’ of the gene of interest, resulting in excision of the CORE6 and knock-in modules and leaving in the gene of interest [51]. The first CORE6 construct was amplified from the plasmid CORE6 [51] by PCR using the primers CORE6w50bpA_LacZ_forw and CORE6w50bpB_LacZ_rev containing 50 bp upstream and downstream of the *lacZ* gene in YCpMmyc1.1, respectively. The DreamTaq DNA Polymerase (ThermoFischer Scientific) was used according to the manufacturer’s specifications, with an annealing temperature of 54°C and elongation time of 2 minutes and 40 seconds.

The second construct was generated in two steps: First the primers 3’Kan_forw and 3’Kan_50 bpA_LacZ_rev were used to amplify the 3’Kan sequence from plasmid pFA6aKanMx4 [51] and mCherry was amplified from a mCherry containing pUC57 plasmid generated by GenScript using the primers mCherry_50 bpA_lacZ_forw and mCherry_50 bpB_lacZ_rev. Both PCRs were performed using DreamTaq DNA Polymerase (ThermoFischer Scientific) with an annealing temperature of 54°C and elongation time of 1 minute. Then the two fragments were joined and amplified in a fusion PCR using the primers 3’Kan_forw and mCherry_50 bpB_lacZ_rev, again using DreamTaq. The Fusion PCR had two stages: first 5 cycles with an annealing temperature of 45°C kept for 3 minutes and an elongation time of 3 minutes, followed by 30 cycles with an annealing time of 60°C (for 30 seconds) and an elongation time of 2 minutes 30 seconds.

Yeast VL6-48N containing the GM12 genome YCpMmyc1.1 was transformed with the CORE6 construct using the Lithium Acetate method [54] and selected on synthetic minimal medium containing glucose (SD) lacking histidine and uracil. Correct insertion of the construct was confirmed via PCR using the primers CORE3-Ura-DG2-F and New_LacZ_downstream.

A second round of transformation using the yeast strain now containing CORE6 instead of *lacZ* was performed with the second construct containing mCherry and yeast transformants were selected on YPD agar containing 0.2 mg/mL geneticin (G418; Sigma-Aldrich, catalogue number A1720). The colonies were replica plated to YPGal plates to induce expression of the I-SceI gene and a double strand break at the 18 bp recognition site for I-SceI resulting in enhanced homologous recombination between the two 50 bp homologous regions upstream of the inserted construct and upstream of the mCherry sequence. Finally, the yeast colonies were grown on 5-FOA for Uracil counter selection. Correct replacement of the lacZ with the mCherry-encoding gene in the YCpMmyc1.1 was confirmed via PCR using the primers New_LacZ_upstream and New_LacZ_downstream. The edited GM12::YCpMmyc1.1-mCherry gene-encoding genome was transplanted into *M. capricolum* subsp. *capricolum* ∆RE [16] and correct clones were confirmed by PCR and Sanger sequencing of the fragment.

### Isolation of ruminant peripheral blood mononuclear cells (PBMCs)

Blood from adult goats and cows, aged 1–3 years, was collected via jugular vein puncture. Caprine blood was collected into bottles containing Alsever’s solution (1.55 mM of C_6_H_12_O_6_ × H_2_O, 408 mM of Na_3_C_6_H_5_O_7_ × 2H_2_O, 1.078 mM of NaCl, and 43 mM of C_6_H_8_O_7_, pH 6.2), with two parts of blood mixed with one part of Alsever’s solution. Bovine blood was collected into vacutainer EDTA tubes (Becton Dickinson). PBMCs were isolated using a standard protocol and described recently [18].

### Stimulation of peripheral blood mononuclear cells by mycoplasmas

Twelve-well flat-bottom plates (TPP, Switzerland) were seeded with 2 × 10^6^ freshly isolated caprine or bovine PBMCs per well in 1 mL Dulbecco’s modified Eagle’s medium (DMEM) (Life Technologies) supplemented with 10% fetal bovine serum (Life Technologies). Preceding the exposure to mycoplasma, freshly isolated ruminant PBMCs were incubated at 38.5°C (ruminant physiological temperature) or 37°C (commonly used in laboratory temperature) under 5% CO_2_ atmosphere incubator for 1–2 hours.

PBMC stimulations with live or heat-inactivated GM12 and its isogenic mutants were done at a multiplicity of infection (MOI) of 100, for 16 hours (caprine PBMCs) or 18 hours (bovine PBMCs) at 38.5°C. After 14 hours of stimulation, 50 μl of supernatant was collected from the different samples and stored at -20°C for subsequent cytokine measurement (multiplex immunoassay). Then, brefeldin A (10 μg/mL) (ThermoFisher) was added to the wells to block PBMCs’ secretion of cytokines and the incubation was continued for another 4 hours, to allow the FCM intracellular staining for measurement of *de novo* cytokine synthesis.

### Cytotoxicity assay

Cell viability was measured using LIVE/DEAD Fixable Yellow Dead Cell Stain kit (Invitrogen, ThermoFisher Scientific) or PE Annexin V Apoptosis Detection kit with 7-AAD (BioLegend) following manufacturer’s instructions with a few modifications. Briefly, the harvested PBMCs were washed in PBS (Dulbecco’s Phosphate Buffered Saline, Gibco) and then stained with fixable LIVE/DEAD stain (1:1000) and incubated on ice in the dark for 20 minutes. Afterwards, the cells were washed in PBS supplemented with 2% fetal bovine serum (Life Technologies) and then resuspended in Annexin V (1:20) and 7-AAD (1:20). Incubation was done at room temperature in the dark for 15 minutes followed by immediate flow cytometer acquisition.

### Multiparameter flow cytometry assay

The caprine and bovine immune cell subtypes were identified by flow cytometry (FCM) using a modified 3-step, 7-color staining protocols for caprine PBMCs and 7-step, 11/12-color staining protocols for bovine PBMCs [18] with a few modifications. A few modifications with respect to antibodies and their fluorophores have been employed. The CD3 specific antibody was labelled using the Zenon Alexa Fluor 568 labelling kit and instead of the CD44 antibody reported previously we employed the antibody CD45RO, clone IL-A116. All information of the antibodies used are listed in S11 Table. FCM acquisitions were performed on a Cytek Aurora (Cytek Biosciences) using the SpectroFlo software with autofluorescence extraction and further analyzed with FlowJo 10.8.1 (TreeStar).

Caprine monocytes were identified as being surface marker CD14-positive and phenotypes were determined by activation/maturation marker expression (MHC-II, MHC-I, and CD25) after live cell gating (S13A and S13B Fig).

Subpopulations of bovine PBMCs, monocytes (classical, intermediate, and non-classical), dendritic cells (conventional type 1; cDC1 and type 2; cDC2 and plasmacytoid; pDC), γδ T cells, NK cells, CD4^+^ and CD8^+^ T cells, and B cells, were characterized as published recently [18].

For the fold-change analysis of activation/maturation marker following stimulation (stimulation index), the mean fluorescence intensity (MFI) measured in stimulated samples for a given animal was normalized to the MFI measured in unstimulated sample from that same animal.

### Multiplex immunoassay

The supernatants to be investigated for the presence of cytokines/chemokines were collected and immediately stored at -20°C until use. None of the samples investigated was stored more than three months after collection. The cytokines/chemokines were quantified using a magnetic bead-based multiplex immunoassay MILLIPLEX MAP Bovine Cytokine/Chemokine Panel 1 kit (including IFN-γ, IL-1α, IL-1β, IL-4, IL-6, IL-8, IL-10, IL-17A, MIP-1α (CCL3), IL-36RA, IP-10 (CXCL10), MCP-1 (CCL2), MIP-1β (CCL4), TNF-α, and VEGF-A) (Merck/Sigma) following the manufacturer’s instructions. Samples were acquired on a Luminex xMAP INTELLIFLEX including an xPONENT Software version 4.2 software (Luminex) and further analyzed with Belysa software.

### Generation of caprine monocyte-derived macrophages (MDMs)

Monocytes were purified from the PBMCs using MACS separation (Miltenyi Biotec, Bergisch Gladbach) by employing a CD14-specific monoclonal antibody (mAb), Clone CAM36A; mouse IgG1 KingFisher Biotech) (S13C Fig). The protocol for monocyte-derived macrophages (MDMs) generation/differentiation was adapted from a published protocol [37]. Briefly, the isolated monocytes were seeded at a density of 2 × 10^5^ cells/mL DMEM medium+GlutaMAX (Gibco, ThermoFisher Scientific) supplemented with 10% FBS (Gibco, ThermoFisher Scientific), 1% penicillin-streptomycin (10,000 U/mL, from Gibco) and 60 ng caprine granulocyte macrophage colony-stimulating factor (GM-CSF, KingFisher Biotech) per well in 12-well flat-bottom plates (TPP, Switzerland). The cells were cultured at 38.5°C with 5% CO_2_ for three days, then, washed twice with pre-warmed 1x PBS, freshly supplemented DMEM media was added and the cells were incubated for another four days before functional assay were initiated after day seven.

### Primary caprine MDMs stimulation with mycoplasmas

Before starting the functional assay at day eight, the supplemented DMEM medium of MDM cultures was replaced with fresh DMEM supplemented with 10% FBS (without recombinant GM-CSF). MDMs stimulations with GM12 and its isogenic mutants were done at a MOI of 100, at 38.5°C for 6, 24, and 48 hours. Exposed MDMs were then observed by a light microscopy (Eclipse TS100, Nikon) and pictures were taken at 24 hours. Supernatants were collected at 6, 24 and 48 hours p.i. and stored at -20°C for subsequent quantifications of cytokines/chemokines. MDMs were detached by adding twice 10 µL of 5 mM of EDTA solution for 10 minutes, the MDMs were harvested and stained using the LIVE/DEAD Fixable Yellow Dead Cell Stain kit (Invitrogen, ThermoFisher Scientific) and different antibodies to quantify activation/maturation of MHC-II, MHC-I, and CD25, see S11 Table.

### Gentamicin protection assay

Gentamicin protection assay was performed in a similar way to the caprine MDMs stimulation described above. In contrast to the latter MDMs were exposed to GM12 and treated with 500 µg/mL gentamicin (Sigma) to kill extracellular mycoplasmas. Briefly, 2 × 10^5^ MDMs/well were washed three times with PBS and fresh DMEM was added. Then, MDMs were exposed to GM12 at a MOI of 100, and incubated for 20 hours in a 5% CO_2_ environment at 38.5°C. Afterwards the supernatants were collected to measure CFUs before gentamicin treatment, MDMs were washed twice with pre-warmed PBS and DMEM medium with 500 μg/mL gentamicin was added. Negative controls did not contain gentamicin. Then, the cells were incubated for another four hours. Supernatants were collected and bacterial load was measured by determination of CFU. MDMs were then washed three times with pre-warmed PBS to rinse off gentamicin and MDMs were tested for the presence of internalized live mycoplasmas. To this aim, MDMs were lysed using 1 mL of H_2_O for 20 seconds and serial dilutions of the lysates were plated on SP5 agar plates to determine the number of live mycoplasmas using CFU counts.

### Measurement of MDM-*Mycoplasma* interaction

The MDMs (2 × 10^5^ cells/well in 12-well plate) were washed three times with PBS, afterwards fresh DMEM was added. Then MDMs were exposed to GM12::YCpMmyc1.1-mCherry at a MOI of 100 for 48 hours at 38.5°C and 5% CO_2_. The supernatants were collected by aspiration and the MDMs were harvested, stained using the LIVE/DEAD Fixable Yellow Dead Cell Stain kit (Invitrogen, ThermoFisher Scientific) and investigated for their levels of fluorescence by FCM analysis after detachment as described above. In parallel, fluorescence microscopy images were taken with a Evos FL Auto 2 microscope (Invitrogen) to obtain merged visualization of GM12::YCpMmyc1.1-mCherry (Texas red fluorescent channel) and MDMs (brightfield).

### Confocal microscopy

MDMs infected with mycoplasmas were processed for confocal microscopy at 6 and 24 hours p.i. on coverslips. Briefly, coverslips were washed twice with PBS, fixed with 4% PFA for 10 min, washed twice with PBS and then incubated with the primary antibody (Rab7-IgG2b, 1:200) for 20 min on ice, washed twice with PBS. Then, the coverslips were incubated with the secondary antibody (Alexa Fluor 488 goat anti-mouse IgG2b; Invitrogen; 1:500 and phalloidin labeling probes-Alexa Fluor 647; Invitrogen; 1:200) for 20 min on ice. Afterwards coverslips were washed twice with PBS and stained nuclei with DAPI (1:5000) for 10 min at room temperature. Finally, coverslips were mounted with DAKO mounting solution and stored overnight at 4°C in the dark. Images were collected using Nikon Ti-2 cicero spinning disk confocal microscope (Nikon). Image analysis and processing was performed with NIS-Elements Advanced Research laboratory image analysis system (Nikon).

### RNA-seq of caprine PBMCs

Total RNA from caprine PBMCs incubated with mycoplasmas at 38.5°C with 5% CO_2_ for up to 16 hours was extracted using the TRIzol reagent according to manufacturer’s instruction. The RNA quantification, library preparation and sequencing were performed by Novogene (Novogene Co., Ltd., Muchen, Germany). Briefly, the quantity and quality of the total RNA sample was determined by Nanodrop and Agilent 5400. Messenger RNA (mRNA) was enriched and purified from total RNA using poly-T oligo-attached magnetic beads. Following fragmentation, the first-strand cDNA was synthesized using random hexamer primers followed by the second-strand cDNA synthesis. The library was prepared through a series of steps including end repair, A-tailing, adapter ligation, size selection, amplification, and purification. The library quality was assessed using Qubit for quantification and a bioanalyzer for size distribution analysis. Quantified libraries were pooled and sequenced on the Illumina platform - NovaSeq X Plus Series 25B (PE150 strategy) based on effective library concentration and desired data volume.

### Analysis of RNA-seq data

RNA-seq data analysis was performed on the Linux Cluster of the Interfaculty Bioinformatics Unit at the University of Bern. The quality of the RNA-seq data was evaluated using FastQC [55] (version 0.12.1). Adapter sequences were removed and reads with a Phred base quality threshold below 15 (Q < 15) or shorter than 15 bp were filtered out using FASTp [56] (version 0.22.0). The cleaned reads were then aligned to the *Capra hircus* (goat) reference genome (ARS1.2, GCF_001704415.2) [57] using STAR [58] (version 2.7.11b). FeatureCounts [59] (version 2.0.6) was employed to quantify the number of reads assigned to each gene. Read quality and alignment was summarized and evaluated with MultiQC [60] (version 1.22.2). Differential gene expression analysis between experimental groups was conducted using the Bioconductor package DESeq2 [61]. All analyses were carried out in R (version 4.4). Raw counts were normalized using DESeq2’s internal method. For unbiased analysis, stimulated samples of each subset were compared to corresponding control samples. Differentially expressed genes (DEGs) were identified using contrasts between experimental groups, and log_2_ fold changes were shrinkage-adjusted using the ashr method. Genes with adjusted p value < 0.05 and |log_2_FC| > 2 were considered significant. Functional enrichment analysis of all DEGs were accomplished on the basis of the gene ontology (GO) database using DAVID (Database for Annotation, Visualization and Integrated Discovery) [62,63].

### Bacterial RNA extraction and quantitative reverse transcriptase PCR (qRT-PCR)

*Mycoplasma* cultures from different growth conditions, as described above, were harvested by centrifugation at 12,400 × *g* for 2 minutes. The pellet was washed with 1 mL PBS, and subsequently total RNA was extracted using the RNAqueous Phenol-free Total RNA Isolation Kit (Thermo Fisher Scientific). RNA concentration was determined employing a Biospectrometer (Eppendorf). Residual genomic DNA was removed using the TURBO DNA-free Kit (Thermo Fisher Scientific), and DNA removal was confirmed by the absence of PCR amplicons targeting the house keeping gene *gyrB* [64] by conventional PCR (S10 Table).

Two-step qRT-PCR was used to quantitatively measure prokaryotic gene expression using the iScript Reverse Transcription Supermix for qRT-PCR (Bio-Rad, USA) and a QuantStudio 5 Real-Time PCR system with QuantStudio Design and Analysis SE Software version v1.5.2 (Applied Biosystems). All primer pairs were designed using NCBI PrimerBlast (https://www.ncbi.nlm.nih.gov/tools/primer-blast/). The primer sequences are shown in S10 Table. Amplification of the *glf* gene [16] was conducted in duplicate in a 10 µL reaction volume containing 5 µL of SsoFast EvaGreen Supermix (Thermo Fisher Scientific), 2 µL (2 ng) of cDNA, 0.4 µL of each primer (10 µmol/L), and 2.2 µL of distilled water. The thermal cycling conditions were as follows: 95°C for 30 seconds, followed by 40 cycles of 95°C for 15 seconds and 60°C for 30 seconds. A melting curve analysis was performed from 65°C to 95°C, increasing 0.1°C per step. The genes *ftsZ*, *rpoA*, and *gyrB* were used as reference controls. Relative gene expression levels were calculated using the 2^−ΔΔCt^ method, where ΔΔCt = (Ct_target – Ct average of three reference genes)_38.5°C or 41°C − (average Ct_target gene – average Ct of three reference genes)_ 37°C at high nutrient conditions [65,66].

### Statistical analysis

Statistical analysis was done using the GraphPad Prism 9 software (GraphPad software, La Jolla, CA, USA). To determine significant differences between groups, paired t tests or one-way repeated measure ANOVA followed by Tukey’s post hoc test were used, as appropriate. A p value < 0.05 was considered statistically significant (*p < 0.05, **p < 0.01, ***p < 0.001, and ****p < 0.0001).

## Supporting information

S1 Fig qRT-PCR relative expression levels of *glf* gene in GM12 cultured in high-nutrient versus low-nutrient medium at different temperatures, 38.5°C and 41°C for 18 hours. *ftsZ*, *gyrB* and *rpoA* were used as a reference for the calculation of relative expression levels. The normalized expression levels were calculated by using 2^−ΔΔCt^ method. Data represent the mean (three biological replicates), and error bars represent the standard deviation.
(TIF)

S2 Fig Doubling times of GM12, GM12::YCpMmyc1.1, GM12::YCpMmyc1.1-Δ68, and GM12::YCpMmyc1.1-Δ*glf* determined at 38.5°C. The values represent the mean with SD of three independent biological replicates.
(TIF)

S3 Fig Confocal micrographs of caprine monocyte-derived macrophages (MDMs) infected with *Mycoplasma mycoides* subsp. *capri* strain GM12. Immunofluorescence images present MDMs stimulated or unstimulated with mycoplasmas at 6 and 24 hours. Nucleic acids of mycoplasmas and MDMs were stained with DAPI (dark blue). Membranes were stained with phalloidin (far-red) and late endosome with anti-Rab7 antibody (green). Yellow arrowheads indicate internalized mycoplasmas.
(TIF)

S4 Fig Cellular viability of freshly isolated peripheral blood mononuclear cells (PBMCs) exposed to GM12 and its isogenic mutants (GM12::YCpMmyc1.1, GM12::YCpMmyc1.1-Δ68 and GM12::YCpMmyc1.1-Δ*glf*) 16 hours post-infection and analyzed by flow cytometry. Percentages of viable cells upon LIVE/DEAD staining on (**A**) caprine PBMCs and (**B**) bovine PBMCs. (**C, D**) The cell viability of caprine PBMCs was determined by Annexin-V and 7-AAD staining. For all plots, Annexin-V, PE versus 7-AAD showing cells alive (lower left), early apoptotic (upper left), late apoptosis/dead (upper right), and necrotic (lower right). (**E**) MHC-II expression on monocytes in non-apoptotic (Annexin V^-^/7-AAD^-^), early apoptotic (Annexin V^+^/7-AAD^-^) and late apoptotic (Annexin V^+^/7-AAD^+^) subpopulations exposed to different mycoplasmas for 16 hours. Data are presented as mean ± standard deviation. Stars indicate significance levels (*p < 0.05, **p < 0.01, and ***p < 0.001). Unstimulated PBMCs were used as a negative control.
(TIF)

S5 Fig Antibody staining showing strong signal for bovine cells compared to caprine cells. Caprine monocytes, γδ T cells and NK cells were incubated with the following primary antibodies: anti-CD14 (Clone CAM66A, IgM, undiluted; secondary antibody: anti-mouse IgM, BV786), anti-WC1-FITC (Clone 19.19, IgG1, undiluted), and anti-CD335 (Clone EC1.1, IgG1, 1:20 dilution; secondary antibody: anti-mouse IgG1, AF546), respectively. Bovine monocytes, γδ T cells and NK cells were incubated with the following primary antibodies: anti-CD14 (Clone CAM36A, IgG1, 1:800 dilution; secondary antibody: anti-mouse IgG1-PE-Cy7, Clone RMG1-1, rat IgG, 1:640 dilution), anti-WC1 (Clone CC15, IgG2a, 1:800 dilution; secondary antibody: anti-mouse IgG2a-Super Bright 780, Clone m2a-15F8, rat IgG1, 1:40 dilution), and anti-CD335 (Clone AKS1, IgG1, 1:100 dilution; secondary antibody: anti-mouse IgG1, AF700), respectively.
(TIF)

S6 Fig Adhesion of peripheral blood mononuclear cells (PBMCs) on colonies of GM12 and GM12::YCpMmyc1.1-Δ*glf.* (TIF)

S7 Fig Ruminant cytokines secreted after incubation of peripheral blood mononuclear cells (PBMCs) with different mycoplasmas. Data are presented as mean ± standard deviation. Stars indicate significance levels (*p < 0.05 and **p < 0.01). Data was subtracted with no stimulation. (**A**) Cytokines secreted by caprine PBMCs. Of note for MIP-1α, IP-10, IL-36RA and IL-4, some PBMCs stimulated with mycoplasmas gave values below detection limit. MIP-1β and MCP-1 were below the detection threshold in all samples. (**B**) Cytokines secreted by bovine PBMCs. Of note for MIP-1β and MCP-1, PBMCs stimulated with GM12 gave values below detection limit. IL-4 and VEGF-A were below the detection threshold in all samples.
(TIF)

S8 Fig Functional enrichment analysis of differentially expressed genes (DEGs) compared between GM12::YCpMmyc1.1-Δ*glf* and GM12 at 6 hours (A) and 16 hours post infection (B). Gene ontology (GO) term of DEGs were performed using DAVID bioinformatics program.
(TIF)

S9 Fig Transcripts per million (TPM) expression values of the representative program cell death genes in caprine peripheral blood mononuclear cells (PBMCs) response to mycoplasmas at 6 and 16 hours post infection. Dot plots of the number of TPM associated with apoptosis, pyroptosis, and necroptosis genes. RM one way ANOVA, Tukey’s multiple comparisons test. *p < 0.05, **p < 0.01 and ****p < 0.0001.
(TIF)

S10 Fig Effect of different growth conditions to colonies of eight different *Mycoplasma mycoides* subsp. *capri* strains. Effect of 38.5°C and 41°C on low nutrient medium agar after 4 days of incubation. The colonies were transferred onto a nitrocellulose membrane and Periodic Acid-Schiff (PAS) stained.
(TIF)

S11 FigInfluence of physiological temperature on ruminant primary peripheral blood mononuclear cells (PBMCs) stimulated with *Mycoplasma mycoides* subsp. *capri* GM12.Ruminant PBMCs were stimulated with GM12 at a multiplicity of infection (MOI) of 100, for 16 hours (caprine PBMCs) or 18 hours (bovine PBMCs). The stimulations were run in parallel at 37°C or 38.5°C (ruminant body temperature). (**A, B**) Percentage of viable cells upon Live/Dead staining at 16 hours for (**A**) caprine PBMCs and (**B**) bovine PBMCs. (**C-F**) Modulation of activation markers on (**C**) caprine monocytes (MHC-I, MHC-II, CD25), (**D**) bovine γδ T cells (CD25) (**E**) monocytes, (**F**) dendritic cells (MHC-II, CD25, CCR7). Data are presented as mean± standard deviation (*p < 0.05, **p < 0.01, ***p < 0.001, and ****p < 0.0001).(TIF)

S12 Fig Effect of different growth conditions of mycoplasmas on host cell viability. Cytotoxic effect of mycoplasmas cultured in high-nutrient versus low-nutrient media at different temperatures, 38.5°C and 41°C on bovine peripheral blood mononuclear cells (n = 8 cattle) for 16 hours, analyzed by flow cytometry. Data are presented as mean ± standard deviation (**p < 0.01, and ***p < 0.001).
(TIF)

S13 Fig Gating strategy of ruminant caprine peripheral blood mononuclear cells (PBMCs) by FACS analysis. (**A**) Viability of caprine PBMCs was assessed using a LIVE/DEAD staining. The percentage of viable cells was calculated as: % of alive cells in all events = (number of LIVE/DEAD negative/ all events) × 100. (**B**) Gating strategy used to identify monocytes. Representative flow cytometry plots from the caprine PBMCs of one of the study. The lymphocytes were live gated during acquisition using the side (SSC) and forward (FCS) scatter plot display. Furthermore, by using the negative gating strategy, live/dead negative lymphocyte population was identified. The monocyte population (CD14^+^) was identified by MHC-II-positive population. (**C**) Purities of enriched monocytes.
(TIF)

S1 Table Comparison of cytokine secretion of caprine peripheral blood mononuclear cells stimulated with GM12 and its isogenic mutants at 38.5°C for 16 hours. (XLSX)

S2 Table Comparison of cytokine secretion of bovine peripheral blood mononuclear cells stimulated with GM12 and its isogenic mutants at 38.5°C for 16 hours. (XLSX)

S3 Table 246 differentially expressed genes in caprine peripheral blood mononuclear cells stimulated with GM12 and GM12::YCpMmyc1.1-Δ*glf* recorded 6 hours post-infection. The data show 222 up-regulated genes and 24 down-regulated genes in GM12::YCpMmyc1.1-Δ*glf*.
(XLSX)

S4 Table 748 differentially expressed genes in caprine peripheral blood mononuclear cells stimulated with GM12 and GM12::YCpMmyc1.1-Δ*glf* recorded 16 hours post-infection. The data show 390 up-regulated genes and 358 down-regulated genes in GM12::YCpMmyc1.1-Δ*glf*.
(XLSX)

S5 Table Functional analysis of differentially expressed genes (246 DEGs) of peripheral blood mononuclear cells incubated GM12::YCpMmyc1.1-Δ*glf* in comparison to GM12 determined 6 hours post-infection. (XLSX)

S6 Table Functional analysis of differentially expressed genes (748 DEGs) of peripheral blood mononuclear cells incubated GM12::YCpMmyc1.1-Δ*glf* in comparison to GM12 determined 16 hours post-infection. (XLSX)

S7 Table Comparison of viable cells upon LIVE/DEAD staining of bovine PBMCs stimulated with GM12 in different culture condition for 16 hours. (XLSX)

S8 Table *Mycoplasma* strains used in this study. (XLSX)

S9 Table *Mycoplasma* media used in this study. (XLSX)

S10 Table Oligonucleotides used in this study. (XLSX)

S11 Table Antibodies and flow cytometry reagents used in this study. (XLSX)

## References

[1] Di Teodoro G, Marruchella G, Di Provvido A, D’Angelo AR, Orsini G, Di Giuseppe P, et al. Contagious Bovine Pleuropneumonia: A Comprehensive Overview. Vet Pathol. 2020;57(4):476–89. doi: 10.1177/0300985820921818 32390522

[2] Thiaucourt F, Bölske G. Contagious caprine pleuropneumonia and other pulmonary mycoplasmoses of sheep and goats. Rev Sci Tech. 1996;15(4):1397–414. doi: 10.20506/rst.15.4.990 9190020

[3] DaMassa AJ, Wakenell PS, Brooks DL. Mycoplasmas of goats and sheep. J Vet Diagn Invest. 1992;4(1):101–13. doi: 10.1177/104063879200400126 1554763

[4] DaMassa AJ, Brooks DL, Adler HE. Caprine mycoplasmosis: widespread infection in goats with Mycoplasma mycoides subsp mycoides (large-colony type). Am J Vet Res. 1983;44(2):322–5. doi: 10.2460/ajvr.1983.44.02.322 6338774

[5] Jores J, Ma L, Ssajjakambwe P, Schieck E, Liljander A, Chandran S, et al. Removal of a Subset of Non-essential Genes Fully Attenuates a Highly Virulent Mycoplasma Strain. Front Microbiol. 2019;10:664. doi: 10.3389/fmicb.2019.00664 31001234 PMC6456743

[6] Buttery SH, Plackett P. A specific polysaccharide from Mycoplasma mycoides. J Gen Microbiol. 1960;23:357–68. doi: 10.1099/00221287-23-2-357 13689482

[7] Buttery SH, Lloyd LC, Titchen DA. Acute respiratory, circulatory and pathological changes in the calf after intravenous injections of the galactan from Mycoplasma mycoides subsp. mycoides. J Med Microbiol. 1976;9(4):379–91. doi: 10.1099/00222615-9-4-379 794475

[8] Browning GF, Marenda MS, Noormohammadi AH, Markham PF. The central role of lipoproteins in the pathogenesis of mycoplasmoses. Vet Microbiol. 2011;153(1–2):44–50. doi: 10.1016/j.vetmic.2011.05.031 21684094

[9] Chambaud I, Wróblewski H, Blanchard A. Interactions between mycoplasma lipoproteins and the host immune system. Trends Microbiol. 1999;7(12):493–9. doi: 10.1016/s0966-842x(99)01641-8 10603485

[10] Arfi Y, Minder L, Di Primo C, Le Roy A, Ebel C, Coquet L, et al. MIB-MIP is a mycoplasma system that captures and cleaves immunoglobulin G. Proc Natl Acad Sci U S A. 2016;113(19):5406–11. doi: 10.1073/pnas.1600546113 27114507 PMC4868467

[11] Blötz C, Stülke J. Glycerol metabolism and its implication in virulence in Mycoplasma. FEMS Microbiol Rev. 2017;41(5):640–52. doi: 10.1093/femsre/fux033 28961963

[12] Gaurivaud P, Lakhdar L, Le Grand D, Poumarat F, Tardy F. Comparison of in vivo and in vitro properties of capsulated and noncapsulated variants of Mycoplasma mycoides subsp. mycoides strain Afadé: a potential new insight into the biology of contagious bovine pleuropneumonia. FEMS Microbiol Lett. 2014;359(1):42–9. doi: 10.1111/1574-6968.12579 25123820

[13] Bertin C, Pau-Roblot C, Courtois J, Manso-Silván L, Thiaucourt F, Tardy F, et al. Characterization of free exopolysaccharides secreted by Mycoplasma mycoides subsp. mycoides. PLoS One. 2013;8(7):e68373. doi: 10.1371/journal.pone.0068373 23869216 PMC3711806

[14] Totté P, Puech C, Rodrigues V, Bertin C, Manso-Silvan L, Thiaucourt F. Free exopolysaccharide from Mycoplasma mycoides subsp. mycoides possesses anti-inflammatory properties. Vet Res. 2015;46:122. doi: 10.1186/s13567-015-0252-6 26490663 PMC4618858

[15] Lartigue C, Vashee S, Algire MA, Chuang R-Y, Benders GA, Ma L, et al. Creating bacterial strains from genomes that have been cloned and engineered in yeast. Science. 2009;325(5948):1693–6. doi: 10.1126/science.1173759 19696314

[16] Schieck E, Lartigue C, Frey J, Vozza N, Hegermann J, Miller RA, et al. Galactofuranose in Mycoplasma mycoides is important for membrane integrity and conceals adhesins but does not contribute to serum resistance. Mol Microbiol. 2016;99(1):55–70. doi: 10.1111/mmi.13213 26354009

[17] Jores J, Schieck E, Liljander A, Sacchini F, Posthaus H, Lartigue C, et al. In vivo role of capsular polysaccharide in Mycoplasma mycoides. J Infect Dis. 2019;219(10):1559–63. doi: 10.1093/infdis/jiy713 30541131 PMC6473168

[18] Démoulins T, Yimthin T, Lindtke D, Eggerschwiler L, Siegenthaler R, Labroussaa F, et al. Temperature impacts the bovine ex vivo immune response towards Mycoplasmopsis bovis. Vet Res. 2024;55(1):18. doi: 10.1186/s13567-024-01272-3 38351086 PMC10863263

[19] Démoulins T, Cherbuin JDR, Yimthin T, Eggerschwiler L, Adnan F, Jores J. Beware of host immune responses towards bacteriophages potentially impacting phage therapy. Vet Res. 2025;56(1):170. doi: 10.1186/s13567-025-01600-1 40817079 PMC12357382

[20] Jores J, Baldwin C, Blanchard A, Browning GF, Colston A, Gerdts V, et al. Contagious Bovine and Caprine Pleuropneumonia: a research community’s recommendations for the development of better vaccines. NPJ Vaccines. 2020;5(1):66. doi: 10.1038/s41541-020-00214-2 32728480 PMC7381681

[21] Lartigue C, Valverde Timana Y, Labroussaa F, Schieck E, Liljander A, Sacchini F, et al. Attenuation of a Pathogenic Mycoplasma Strain by Modification of the obg Gene by Using Synthetic Biology Approaches. mSphere. 2019;4(3):e00030-19. doi: 10.1128/mSphere.00030-19 31118296 PMC6531878

[22] Bertin C, Pau-Roblot C, Courtois J, Manso-Silván L, Tardy F, Poumarat F, et al. Highly dynamic genomic loci drive the synthesis of two types of capsular or secreted polysaccharides within the Mycoplasma mycoides cluster. Appl Environ Microbiol. 2015;81(2):676–87. doi: 10.1128/AEM.02892-14 25398856 PMC4277593

[23] Ligasová A, Vydržalová M, Buriánová R, Brůčková L, Večeřová R, Janošťáková A, et al. A New Sensitive Method for the Detection of Mycoplasmas Using Fluorescence Microscopy. Cells. 2019;8(12):1510. doi: 10.3390/cells8121510 31775352 PMC6952905

[24] Bonnefois T, Vernerey M-S, Rodrigues V, Totté P, Puech C, Ripoll C, et al. Development of fluorescence expression tools to study host-mycoplasma interactions and validation in two distant mycoplasma clades. J Biotechnol. 2016;236:35–44. doi: 10.1016/j.jbiotec.2016.08.006 27497759

[25] Kapoor PK, Garg DN, Mahajan SK. Isolation of Mycoplasma mycoides subsp. mycoides (LC variant, Y-Goat) from naturally aborted bovine fetuses. Theriogenology. 1989;32(4):683–91. doi: 10.1016/0093-691x(89)90289-6 16726715

[26] Jores J, Mariner JC, Naessens J. Development of an improved vaccine for contagious bovine pleuropneumonia: an African perspective on challenges and proposed actions. Vet Res. 2013;44(1):122. doi: 10.1186/1297-9716-44-122 24359340 PMC3910389

[27] Labroussaa F, Torres-Puig S, Jores J. Genome transplantation in Mollicutes. In: Gurtler V, Calcutt M, editors. Methods in Microbiology, vol. 52. Elsevier; 2023. p. 3–32.

[28] Gaurivaud P, Baranowski E, Pau-Roblot C, Sagné E, Citti C, Tardy F. Mycoplasma agalactiae Secretion of β-(1→6)-Glucan, a Rare Polysaccharide in Prokaryotes, Is Governed by High-Frequency Phase Variation. Appl Environ Microbiol. 2016;82(11):3370–83. doi: 10.1128/AEM.00274-16 27037120 PMC4959233

[29] Vastel M, Pau-Roblot C, Ferré S, Tocqueville V, Ambroset C, Marois-Créhan C, et al. Capsular Polysaccharide Production in Bacteria of the Mycoplasma Genus: A Huge Diversity of Pathways and Synthases for So-Called Minimal Bacteria. Mol Microbiol. 2024;122(6):866–78. doi: 10.1111/mmi.15325 39473362 PMC11658790

[30] Mallia P, Webber J, Gill SK, Trujillo-Torralbo M-B, Calderazzo MA, Finney L, et al. Role of airway glucose in bacterial infections in patients with chronic obstructive pulmonary disease. J Allergy Clin Immunol. 2018;142(3):815-823.e6. doi: 10.1016/j.jaci.2017.10.017 29310905 PMC6127032

[31] Citti C, Nouvel L-X, Baranowski E. Phase and antigenic variation in mycoplasmas. Future Microbiol. 2010;5(7):1073–85. doi: 10.2217/fmb.10.71 20632806

[32] Hegde S, Hegde S, Spergser J, Brunthaler R, Rosengarten R, Chopra-Dewasthaly R. In vitro and in vivo cell invasion and systemic spreading of Mycoplasma agalactiae in the sheep infection model. Int J Med Microbiol. 2014;304(8):1024–31. doi: 10.1016/j.ijmm.2014.07.011 25129554 PMC4282308

[33] Mulongo M, Prysliak T, Scruten E, Napper S, Perez-Casal J. In vitro infection of bovine monocytes with Mycoplasma bovis delays apoptosis and suppresses production of gamma interferon and tumor necrosis factor alpha but not interleukin-10. Infect Immun. 2014;82(1):62–71. doi: 10.1128/IAI.00961-13 24126524 PMC3911867

[34] Iwasaki A, Foxman EF, Molony RD. Early local immune defences in the respiratory tract. Nat Rev Immunol. 2017;17(1):7–20. doi: 10.1038/nri.2016.117 27890913 PMC5480291

[35] Sterner-Kock A, Haider W, Sacchini F, Liljander A, Meens J, Poole J, et al. Morphological characterization and immunohistochemical detection of the proinflammatory cytokines IL-1β, IL-17A, and TNF-α in lung lesions associated with contagious bovine pleuropneumonia. Trop Anim Health Prod. 2016;48(3):569–76. doi: 10.1007/s11250-016-0994-9 26837619 PMC4766205

[36] Totté P, Bonnefois T, Manso-Silván L. Interactions between Mycoplasma mycoides subsp. mycoides and bovine macrophages under physiological conditions. PLoS One. 2024;19(6):e0305851. doi: 10.1371/journal.pone.0305851 38935768 PMC11210856

[37] Sautter CA, Auray G, Python S, Liniger M, Summerfield A. Phenotypic and functional modulations of porcine macrophages by interferons and interleukin-4. Dev Comp Immunol. 2018;84:181–92. doi: 10.1016/j.dci.2018.01.018 29408047

[38] Sacchini F, Luciani M, Salini R, Scacchia M, Pini A, Lelli R, et al. Plasma levels of TNF-α, IFN-γ, IL-4 and IL-10 during a course of experimental contagious bovine pleuropneumonia. BMC Vet Res. 2012;8:44. doi: 10.1186/1746-6148-8-44 22533922 PMC3378467

[39] Sacchini F, Naessens J, Awino E, Heller M, Hlinak A, Haider W, et al. A minor role of CD4+ T lymphocytes in the control of a primary infection of cattle with Mycoplasma mycoides subsp. mycoides. Vet Res. 2011;42(1):77. doi: 10.1186/1297-9716-42-77 21663697 PMC3148206

[40] Niang M, Rosenbusch RF, Andrews JJ, Kaeberle ML. Demonstration of a capsule on Mycoplasma ovipneumoniae. Am J Vet Res. 1998;59(5):557–62. doi: 10.2460/ajvr.1998.59.5.557 9582956

[41] Tajima M, Yagihashi T, Miki Y. Capsular material of Mycoplasma gallisepticum and its possible relevance to the pathogenic process. Infect Immun. 1982;36(2):830–3. doi: 10.1128/iai.36.2.830-833.1982 6177640 PMC351303

[42] Almeida RA, Wannemuehler MJ, Rosenbusch RF. Interaction of Mycoplasma dispar with bovine alveolar macrophages. Infect Immun. 1992;60(7):2914–9. doi: 10.1128/iai.60.7.2914-2919.1992 1612758 PMC257254

[43] Bolland JR, Simmons WL, Daubenspeck JM, Dybvig K. Mycoplasma polysaccharide protects against complement. Microbiology (Reading). 2012;158(Pt 7):1867–73. doi: 10.1099/mic.0.058222-0 22504437 PMC3542145

[44] Jiang Z, Song F, Li Y, Xue D, Deng G, Li M, et al. Capsular Polysaccharide is a Main Component of Mycoplasma ovipneumoniae in the Pathogen-Induced Toll-Like Receptor-Mediated Inflammatory Responses in Sheep Airway Epithelial Cells. Mediators Inflamm. 2017;2017:9891673. doi: 10.1155/2017/9891673 28553017 PMC5434471

[45] Citti C, Blanchard A. Mycoplasmas and their host: emerging and re-emerging minimal pathogens. Trends Microbiol. 2013;21(4):196–203. doi: 10.1016/j.tim.2013.01.003 23419218

[46] Mwirigi M, Nkando I, Olum M, Attah-Poku S, Ochanda H, Berberov E, et al. Capsular polysaccharide from Mycoplasma mycoides subsp. mycoides shows potential for protection against contagious bovine pleuropneumonia. Vet Immunol Immunopathol. 2016;178:64–9. doi: 10.1016/j.vetimm.2016.07.002 27496744

[47] Rosengarten R, Wise KS. Phenotypic switching in mycoplasmas: phase variation of diverse surface lipoproteins. Science. 1990;247(4940):315–8. doi: 10.1126/science.1688663 1688663

[48] Persson A, Jacobsson K, Frykberg L, Johansson K-E, Poumarat F. Variable surface protein Vmm of Mycoplasma mycoides subsp. mycoides small colony type. J Bacteriol. 2002;184(13):3712–22. doi: 10.1128/JB.184.13.3712-3722.2002 12057968 PMC135138

[49] Labroussaa F, Lebaudy A, Baby V, Gourgues G, Matteau D, Vashee S, et al. Impact of donor-recipient phylogenetic distance on bacterial genome transplantation. Nucleic Acids Res. 2016;44(17):8501–11. doi: 10.1093/nar/gkw688 27488189 PMC5041484

[50] Torres-Puig S, Crespo-Pomar S, Akarsu H, Yimthin T, Cippà V, Démoulins T, et al. Functional surface expression of immunoglobulin cleavage systems in a candidate Mycoplasma vaccine chassis. Commun Biol. 2024;7(1):779. doi: 10.1038/s42003-024-06497-8 38942984 PMC11213901

[51] Chandran S, Noskov VN, Segall-Shapiro TH, Ma L, Whiteis C, Lartigue C, et al. TREC-IN: gene knock-in genetic tool for genomes cloned in yeast. BMC Genomics. 2014;15(1):1180. doi: 10.1186/1471-2164-15-1180 25539750 PMC4407568

[52] Benders GA, Noskov VN, Denisova EA, Lartigue C, Gibson DG, Assad-Garcia N, et al. Cloning whole bacterial genomes in yeast. Nucleic Acids Res. 2010;38(8):2558–69. doi: 10.1093/nar/gkq119 20211840 PMC2860123

[53] Larionov V, Kouprina N, Solomon G, Barrett JC, Resnick MA. Direct isolation of human BRCA2 gene by transformation-associated recombination in yeast. Proc Natl Acad Sci U S A. 1997;94(14):7384–7. doi: 10.1073/pnas.94.14.7384 9207100 PMC23830

[54] Gietz D, St Jean A, Woods RA, Schiestl RH. Improved method for high efficiency transformation of intact yeast cells. Nucleic Acids Res. 1992;20(6):1425. doi: 10.1093/nar/20.6.1425 1561104 PMC312198

[55] FastQC: a quality control tool for high throughput sequence data. [Internet]. 2010. Available from: http://www.bioinformatics.babraham.ac.uk/projects/fastqc

[56] Chen S, Zhou Y, Chen Y, Gu J. fastp: an ultra-fast all-in-one FASTQ preprocessor. Bioinformatics. 2018;34(17):i884–90. doi: 10.1093/bioinformatics/bty560 30423086 PMC6129281

[57] Bickhart DM, Rosen BD, Koren S, Sayre BL, Hastie AR, Chan S, et al. Single-molecule sequencing and chromatin conformation capture enable de novo reference assembly of the domestic goat genome. Nat Genet. 2017;49(4):643–50. doi: 10.1038/ng.3802 28263316 PMC5909822

[58] Dobin A, Davis CA, Schlesinger F, Drenkow J, Zaleski C, Jha S, et al. STAR: ultrafast universal RNA-seq aligner. Bioinformatics. 2013;29(1):15–21. doi: 10.1093/bioinformatics/bts635 23104886 PMC3530905

[59] Liao Y, Smyth GK, Shi W. featureCounts: an efficient general purpose program for assigning sequence reads to genomic features. Bioinformatics. 2014;30(7):923–30. doi: 10.1093/bioinformatics/btt656 24227677

[60] Ewels P, Magnusson M, Lundin S, Käller M. MultiQC: summarize analysis results for multiple tools and samples in a single report. Bioinformatics. 2016;32(19):3047–8. doi: 10.1093/bioinformatics/btw354 27312411 PMC5039924

[61] Love MI, Huber W, Anders S. Moderated estimation of fold change and dispersion for RNA-seq data with DESeq2. Genome Biol. 2014;15(12):550. doi: 10.1186/s13059-014-0550-8 25516281 PMC4302049

[62] Sherman BT, Hao M, Qiu J, Jiao X, Baseler MW, Lane HC, et al. DAVID: a web server for functional enrichment analysis and functional annotation of gene lists (2021 update). Nucleic Acids Res. 2022;50(W1):W216–21. doi: 10.1093/nar/gkac194 35325185 PMC9252805

[63] Huang DW, Sherman BT, Lempicki RA. Systematic and integrative analysis of large gene lists using DAVID bioinformatics resources. Nat Protoc. 2009;4(1):44–57. doi: 10.1038/nprot.2008.211 19131956

[64] Fischer A, Shapiro B, Muriuki C, Heller M, Schnee C, Bongcam-Rudloff E, et al. The origin of the “Mycoplasma mycoides cluster” coincides with domestication of ruminants. PLoS One. 2012;7(4):e36150. doi: 10.1371/journal.pone.0036150 22558362 PMC3338596

[65] Livak KJ, Schmittgen TD. Analysis of relative gene expression data using real-time quantitative PCR and the 2(-Delta Delta C(T)) Method. Methods. 2001;25(4):402–8. doi: 10.1006/meth.2001.1262 11846609

[66] Paksanont S, Sintiprungrat K, Yimthin T, Pumirat P, Peacock SJ, Chantratita N. Effect of temperature on Burkholderia pseudomallei growth, proteomic changes, motility and resistance to stress environments. Sci Rep. 2018;8(1):9167. doi: 10.1038/s41598-018-27356-7 29907803 PMC6004011

